# Electrically switchable dual-focus metalens for depth-extended in vivo photoacoustic imaging of ophthalmic vascular disease

**DOI:** 10.1038/s41377-026-02365-8

**Published:** 2026-09-04

**Authors:** Hongyoon Kim, Eunwoo Park, Jeongwoo Park, Dong Kyo Oh, Jehyeon Shin, Hyeonsu Heo, Chulhong Kim, Junsuk Rho

**Affiliations:** 1 Department of Mechanical Engineering, Pohang University of Science and Technology (POSTECH), Pohang, 37673 Republic of Korea; 2 Department of Convergence IT Engineering, Pohang University of Science and Technology (POSTECH), Pohang, 37673 Republic of Korea; 3 Department of Electrical Engineering, Pohang University of Science and Technology (POSTECH), Pohang, 37673 Republic of Korea; 4 Department of Biomedical Convergence Science and Technology, Kyungpook National University, Daegu, 41566 Republic of Korea; 5 Department of Advanced Bioconvergence, Kyungpook National University, Daegu, 41566 Republic of Korea; 6 Cell and Matrix Research Institute, Kyungpook National University, Daegu, 41944 Republic of Korea; 7 Graduate School of Artificial Intelligence, Pohang University of Science and Technology (POSTECH), Pohang, 37673 Republic of Korea; 8 Department of Medical Science and Engineering, Pohang University of Science and Technology (POSTECH), Pohang, 37673 Republic of Korea; 9 Department of Chemical Engineering, Pohang University of Science and Technology (POSTECH), Pohang, 37673 Republic of Korea; 10 POSCO-POSTECH-RIST Convergence Research Center for Flat Optics and Metaphotonics, Pohang, 37673 Republic of Korea; 11 National Institute of Nanomaterials Technology (NINT), Pohang, 37673 Republic of Korea

**Keywords:** Imaging and sensing, Adaptive optics, Metamaterials, Nanophotonics and plasmonics, Photoacoustics

## Abstract

High-resolution three-dimensional imaging is essential for visualizing fine biological details. Unlike conventional optical imaging, photoacoustic (PA) imaging provides volumetric imaging by capturing time-of-flight ultrasound waves generated from optical absorption within tissues. Optical-resolution photoacoustic microscopy achieves micron-scale lateral resolution through tight optical focusing but suffers from a shallow depth of focus. To address this, non-diffracting beams (e.g., Bessel and needle beams) have been explored, but they introduce high side lobes that reduce the signal-to-noise ratio and distribute laser energy over a larger volume, reducing signal intensity. Here, we present an electrically modulated dual-focus metalens-based photoacoustic microscopy (meta-PAM) system designed to enhance axial coverage (1.2 mm) while preserving high lateral resolution (3.7 μm) in three-dimensional imaging. Our system features a silicon nitride nanostructured metalens that switches between two diffraction-limited focal modes at a visible wavelength without mechanical movement, with focal switching voltages of 0.87 V and 1.03 V and a response time of 250 ms. We demonstrate its effectiveness by imaging a chemically induced corneal burn in rat eyes, successfully monitoring neovascularization with high resolution across the entire eye depth range. Our compact yet effective system provides building blocks for future high-resolution volumetric imaging with an improved signal-to-noise ratio.

## Abstract (web summary)

Electrically tunable dual-focus metalens-based photoacoustic microscopy enables 1.2 mm axial coverage and 3.7 μm resolution, switching focal modes without mechanical motion for high-resolution volumetric imaging with improved signal-to-noise ratio.

## Graphical abstract

**Figure:**
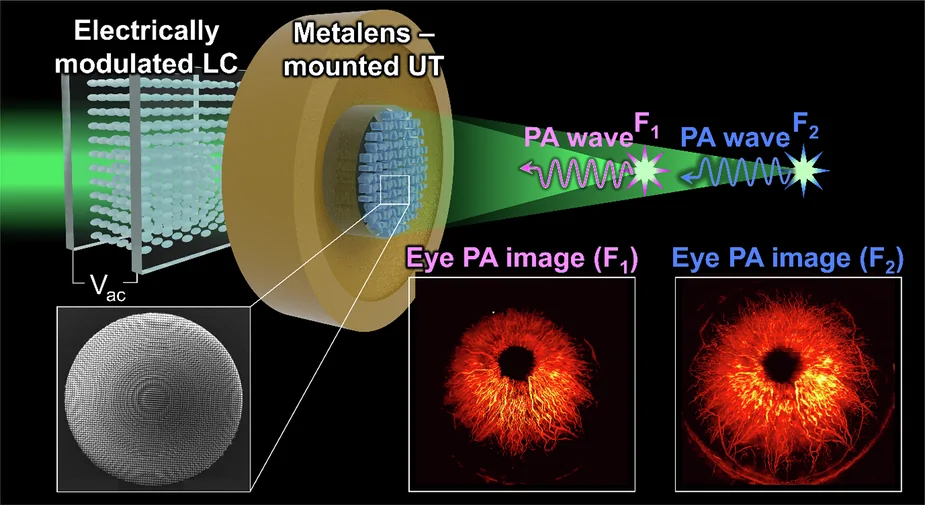

## Introduction

High-resolution three-dimensional (3D) optical imaging is essential in biological research, enabling detailed visualization of biological structures, high-precision diagnostics, and supporting various clinical applications. Optical imaging modalities are particularly advantageous due to their non-ionizing, safe, and non-invasive nature^1–3^. However, most optical imaging techniques generally face inherent resolution limitations in both lateral and axial directions due to diffraction. Achieving high resolution requires a high numerical aperture (NA), but increasing the NA reduces the depth of focus (DOF), restricting high-resolution imaging to a narrow focal range. Therefore, 3D imaging techniques maintain high resolution and a strong signal-to-noise ratio (SNR) near the focal region. However, as the imaging depth increases, both resolution and signal intensity degrade, deteriorating the overall performance of 3D imaging as the distance from the focal point increases.

Photoacoustic microscopy (PAM) is a hybrid modality that utilizes pulsed optical excitation and ultrasonic detection to visualize optical absorption contrast in vivo^4–6^. By exploiting the weak scattering of ultrasound in tissue, PAM achieves high-resolution imaging beyond the ~1 mm optical diffusion limit and provides intrinsic absorption-based contrast for label-free visualization of endogenous chromophores^7–9^. Optical-resolution PAM (OR-PAM) in particular uses a tightly focused laser beam to attain lateral resolution at the optical diffraction limit, enabling the imaging of fine microvascular networks^10,11^. However, this tight optical focusing confers a very shallow DOF^12^. Consequently, the resolution degrades for features outside the focal plane, making it challenging to acquire high-quality 3D images without time-consuming axial re-aligning scans^13^. Thus, the limited DOF imposes a significant constraint on volumetric imaging capability. To address this issue, various optical beam shaping techniques have been introduced. One notable approach involves the use of nondiffracting beams, such as Bessel beams^14,15^ and needle beams^16^, which extend the focal region along the axial direction. These techniques enable the resolution to be maintained over a longer axial range compared to conventional Gaussian beams. However, these techniques suffer from the generation of undesired signals due to high side lobes and experience a significant reduction in signal intensity as the light extends axially, degrading the SNR^17^.

Metasurfaces, composed of materials with artificially designed subwavelength-scale nanostructures, offer promising solutions to address these challenges by enabling precise control over light propagation and wavefront shaping^18–20^. Following the introduction of the generalized Snell’s law, metasurfaces have emerged as a groundbreaking platform in advanced imaging technologies^21^. By carefully engineering the geometry and orientation of nanostructures, meta-atoms, metasurfaces can perform a wide range of optical functions, such as imaging^22–25^, optical hologram^26,27^, color printing^28,29^, and AR/VR^30,31^. Furthermore, the adoption of mass-production fabrication techniques has accelerated the advancement of metasurfaces, paving the way for their widespread commercialization and practical integration into optical technologies^32–35^.

**Fig. 1:**
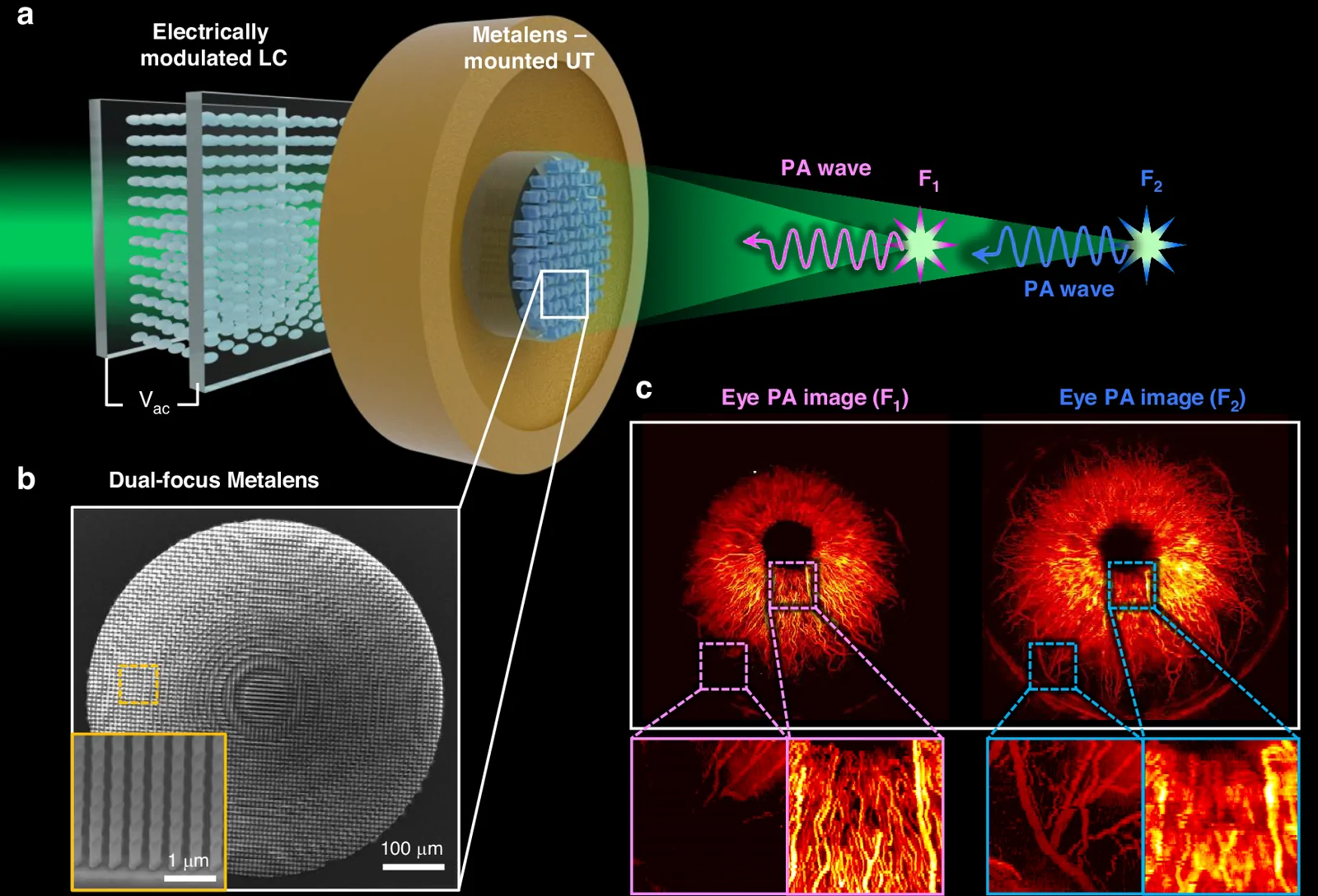
Schematic illustration of the electrically-modulated dual-focus meta-PAM. **a** Schematic of the dual-focus metalens-mounted ultrasound transducer (UT). **b** SEM image of the fabricated dual-focus metalens. **c** In vivo label-free dual-focal PA imaging of the rat eye for each focal mode M_1_ and M_2_

Here, we propose an electrically tunable, dual-focus metalens-based high-SNR PAM system capable of switching focal modes along the axial direction on a millisecond timescale, enabling high-resolution 3D imaging over a broad axial depth range. The dual-focus metalens is composed of silicon nitride (SiN_*x*_) nanostructures designed to focus 532 nm light, at which hemoglobin exhibits strong absorption and thus yields a high PA signal contrast, into two distinct focal lengths, F_1_ and F_2_, for right-handed circularly polarized (RCP) and left-handed circularly polarized (LCP) incidence, respectively. The incident polarization state, and hence the focal length, is controlled by an electrically tunable liquid crystal (LC) cell (Fig. 1a). Therefore, the focal point can be dynamically adjusted by simply controlling the electrical bias applied to the LC cell, without requiring any mechanical movement of optical components. The fabricated dual-focus metalens is mounted and aligned on the ultrasound transducer (UT) to detect the generated PA waves by the optical excitation near the focus (Fig. 1b). The focal length of the UT is placed in the middle of both focal points F_1_, and F_2_ to image PA signals for both focal modes, resulting in high-resolution PA images for both focal modes (Fig. 1c). The extensive imaging regime due to electrically tunable diffraction-limited optical focuses advances PA imaging systems for obtaining high-resolution 3D images with high SNR.

## Results

### Optical properties of silicon nitride

Since PA imaging employs a high-power pulsed laser and detects PA signals arising from optical absorption, the choice of material for the metalens is critical. The material must achieve a high refractive index (*n*) for efficient light confinement with a low extinction coefficient (*k*) to ensure optical robustness under high-power laser exposure, while simultaneously generating low PA signals.

**Fig. 2:**
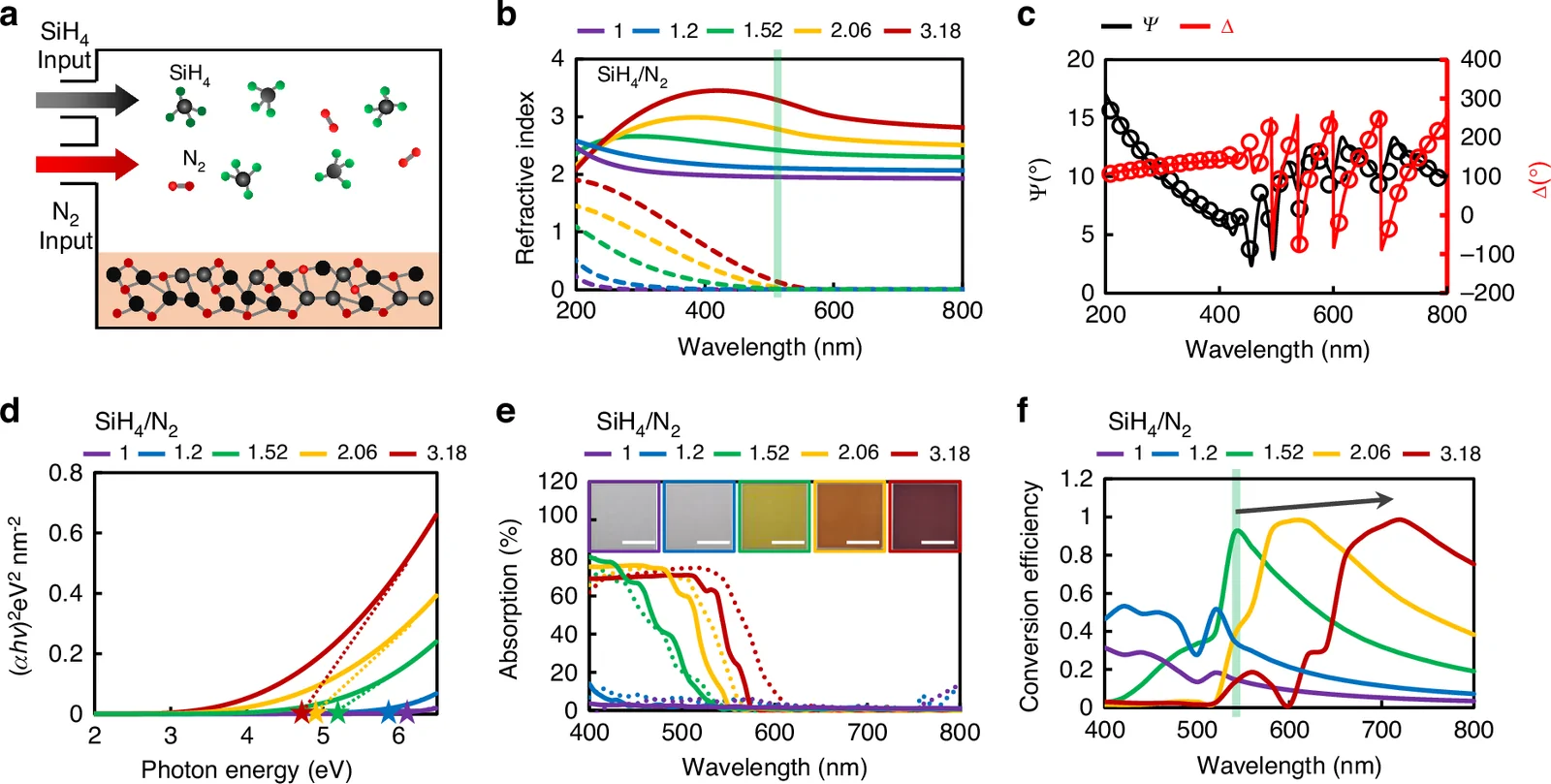
Optical properties of SiN_*x*_ with varying deposition conditions. **a** Schematic of the deposition condition control of SiN_*x*_ film by varying the SiH_4_/N_2_ ratio. **b** The measured refractive index (*n*) (solid line) and extinction coefficient (*k*) (dotted line) for varying SiH_4_/N_2_ ratio from 1 to 3.18. **c** Psi (*Ψ*), and delta (*Δ*) of the optimized SiN_*x*_ film with SiH_4_/N_2_ ratio of 1.52 from ellipsometry measurement (circle) and theoretical prediction by the Tauc–Lorentz model (line). **d** (*αhv*)^2^ and the *hv* relation to obtain the optical bandgap. The star symbols indicate the extracted optical bandgap energy for each SiN_*x*_ film. **e** Measured (dotted line) and simulated (solid line) absorption spectrum for a varying 1000 nm-thick SiN_*x*_ film with a different SiH_4_/N_2_ ratio. The inset shows the fabricated 1000 nm-thick SiN_*x*_ film with a different SiH_4_/N_2_ ratio (Scale bar: 1 cm). **f** Simulated conversion efficiency of a meta-atom with geometric parameters of 340 nm (period), 250 nm (width), and 50 nm (length)

To realize these requirements, we employed bandgap engineering of SiN_*x*_ films deposited by plasma-enhanced chemical vapor deposition (PECVD) using SiH_4_ and N_2_ precursors, with the SiH_4_/N_2_ gas ratio carefully tuned (Fig. 2a). Increasing the SiH_4_ fraction progressively enriches the film with silicon, thereby increasing n due to the higher density of Si-Si bonds and reduced NH bonding. SiN_*x*_ films were deposited at a fixed chamber temperature of 300 °C and pressure of 30 mTorr, while varying the SiH_4_/N_2_ ratio from 1 to 3.2. The refractive indices of the fabricated SiN_*x*_ films were measured using ellipsometry (Fig. 2b). As expected, *n* increased with higher SiH_4_/N_2_ ratios; however, *k* also increased, showing a considerable tradeoff.

Among the deposition conditions, SiH_4_/N_2_ ratio of 1.52 was selected as the optimal material, achieving a high refractive index (*n* = 2.4) with a near-zero extinction coefficient (*k* = 0.0027) at 532 nm wavelength, and the measured psi (*Ψ*) – delta (*Δ*) values well-matched with the calculated values based on the Tauc–Lorentz model (Fig. 2c). In addition, the optical bandgaps of the SiN_*x*_ films were calculated using the Tauc and Davis-Mott equation relation^36^:

1 $${(\alpha {hv})}^{n}=K({hv}-{E}_{{\rm{g}}})$$

where *α* = 4*πk*/*λ* is the absorption coefficient, *hv* is the photon energy, *n* denotes the transition type, *K* is a constant, and *E*_g_ corresponds to the optical bandgap energy. The bandgap energies of SiN_*x*_ films deposited at SiH_4_/N_2_ ratios of 1, 1.2, 1.52, 2.06, and 3.18 were determined to be 6.04, 5.82, 5.17, 4.82, and 4.67 eV, respectively (Fig. 2d). Due to its relatively wide bandgap, the 1000 nm-thick SiN_*x*_ film deposited at a SiH_4_/N_2_ ratio of 1.52 exhibited only ~2% absorption at 532 nm wavelength (Fig. 2e). Note that other 1000 nm-thick SiN_*x*_ films with a higher *n* exhibit stronger absorption at 532 nm due to their larger *k*, and this behavior is consistently reflected in the inherent color of the deposited films.

Moreover, this optimized SiN_*x*_ deposition condition not only suppresses absorption but also maximizes the conversion efficiency at 532 nm wavelength, thereby directly enhancing the focusing efficiency of the metalens (Fig. 2f) (find more information on conversion efficiency in Supplementary Fig. S1, Supporting information).

### Design method of dual-focus metalens

To independently modulate the incident light according to its spin state ($|{\sigma }^{+}{{\rangle }}$ (RCP) or $|{\sigma }^{-}{{\rangle }}$ (LCP)), we combine two phase modulation mechanisms: the propagation phase and the geometric phase, also known as the Pancharatnam–Berry (PB) phase. The transmitted electric field through a single meta-atom can be written as:

2 $${E}_{\mathrm{trans}}=J\left(\theta \right)\cdot |{\sigma }^{\pm }\rangle =\frac{{t}_{{\rm{f}}}+{t}_{{\rm{s}}}}{2}|{\sigma }^{\pm }\rangle +\frac{{t}_{{\rm{f}}}-{t}_{{\rm{s}}}}{2}{e}^{i2\theta }|{\sigma }^{\pm }\rangle$$

where ${t}_{{\rm{f}}}={T}_{{\rm{f}}}{e}^{i{\phi }_{{\rm{f}}}}$, and ${t}_{{\rm{s}}}={T}_{{\rm{s}}}{e}^{i{\phi }_{{\rm{s}}}}$, respectively, and ${\phi }_{{\rm{f}}}$ and ${\phi }_{{\rm{s}}}$ denote the phase retardation along the fast and slow axes. According to Eq. (2), independent phase encoding for the two spin channels can be achieved by combining the propagation phase (first term) with the PB phase (second term), which is a well-established design framework (See detailed information in Supplementary Note 1)^37,38^.

**Fig. 3:**
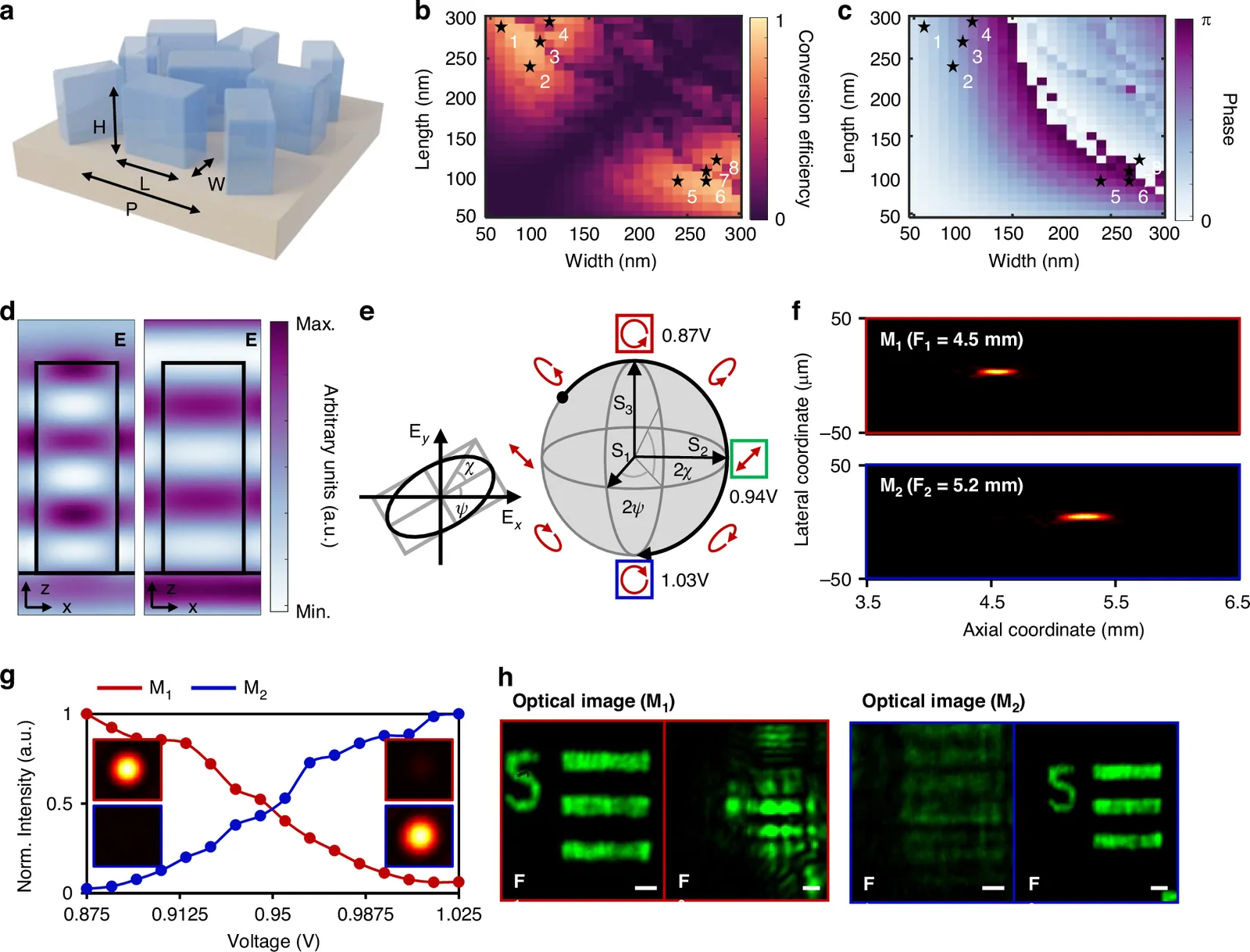
Design methods of dual-focus metalens. **a** Schematic of the designed metalens. **b** Simulated conversion efficiencies of the meta-atoms with various widths (W) and lengths (L). **c** Simulated phase retardation of the meta-atoms with various widths (W) and lengths (L) under *x*-polarized light. **d** Simulated electric field distribution under *x*- (left) and *y*-polarized light (right) of the selected meta-atom at *λ* = 532 nm. **e** Output polarization states passing through the LC when 45° rotated LP is incident. **f** Focal intensity distribution in the *xz*-plane depending on the applied voltage (0.87 V: M_1_, 1.03 V: M_2_). **g** Focal intensity variation on the focal plane. **h** optical images for both modes M_1_ and M_2_ (Scale bars: 10 μm)

To achieve high efficiency in both focal modes, F_1_ and F_2_, we optimize meta-atoms to function as half-wave plates by varying their geometrical parameters, height (H), period (P), length (L), and width (W) (Fig. 3a). Given that PA imaging requires an optical lens to operate underwater to minimize PA signal loss, we design our metalens to achieve optimal efficiency in water (*n* = 1.33). Considering fabrication feasibility, the optimal efficiency is achieved with H = 1000 nm, and P = 340 nm (Fig. 3b). Notably, the conversion efficiencies for RCP and LCP incidences are identical. We further analyze the phase retardation and phase differences for *x*- and *y*-polarized light incidences (Fig. 3c; Supplementary Fig. S2, Supporting Information). We select eight meta-atoms with conversion efficiencies exceeding 70% and phase differences exceeding 0.75π between *x*- and *y*-polarized light, enabling full 2π phase retardation with a π/4 phase increment. The selected meta-atoms and their corresponding dimensions are as follows: [(1) W: 60 nm, L: 290 nm; (2) W: 90 nm, L: 240 nm; (3) W: 100 nm, L: 270 nm; (4) W: 110 nm, L: 300 nm; (5) W: 240 nm, L: 90 nm; (6) W: 270 nm, L: 90 nm; (7) W: 270 nm, L: 100 nm; (8) W: 280 nm, L: 120 nm]. We confirm the half-wave plate characteristic of the selected meta-atom by calculating the electric field under *x*- and *y*-polarized incident light (Fig. 3d). In addition, the conversion efficiency and phase retardation of these meta-atoms, along with their simulated *E*-field distribution, confirm the ability to achieve 2π phase modulation (Supplementary Fig. S3, Supporting Information).

To demonstrate the dual-focus for RCP and LCP light, we design two lens phase maps corresponding to focal lengths of 4.5 mm and 5.2 mm. The phase profiles for each focal length *f* are given by:

3 $$\varphi \left(r\right)=-\frac{2\pi }{\lambda }(f-\sqrt{{r}^{2}+{f}^{2}})$$

where *r* is the radial coordinate of the metalens, and *λ* denotes the wavelength of light. The selected meta-atoms are positioned to represent the average phase value obtained from two phase maps under *x*-polarized light. Due to the limited selection of meta-atoms, the propagation phase is discretized into eight levels, which remain sufficient to achieve a full 2π phase shift. Additionally, since the PB phase introduces equal and opposite phase retardations for LCP and RCP light, we rotate the meta-atoms to match the phase maps for each polarization.

### Optical characterization of the electrically tunable dual-focus metalens

To demonstrate the electrically tunable dual-focus functionality of the fabricated metalens, we integrate LC cells to modulate the polarization states of the incident light. The LC material, 4-cyano-4’pentylbiphenyl (5CB), modifies its effective refractive index along the long axis (extraordinary refractive index, *n*_e_) and short axis (ordinary refractive index, *n*_0_) in response to an applied voltage with a measured response time around 250 ms (Supplementary Fig. S4, Supporting Information)^39^. The resulting effective refractive index change ($\Delta {n}_{\mathrm{eff}}$) is given by:

4 $$\Delta {n}_{\mathrm{eff}}=\frac{{n}_{0}{n}_{e}}{\sqrt{{n}_{0}^{2}{\cos }^{2}\theta +{n}_{e}^{2}{\sin }^{2}\theta }}-{n}_{0}$$

where *θ* is the angle between the rubbing direction and the long axis^39^. By applying different voltages to the LC cells, the LCs realign in response to *V*_ac_, functioning as phase retarders with phase retardation: $\tau ={\int }_{0}^{t}2\pi \Delta {n}_{\mathrm{eff}}(z)/\lambda {dz}$, where *t* is the LC cell thickness. This results in a controlled polarization state rotation of the output light on the Poincaré sphere, depending on the applied voltage (Fig. 3e). At 0.87 V, the output light exhibits RCP, while at 0.94 V, it transitions to 45° linearly polarized light (LP), and at 1.03 V, it becomes LCP. The detailed polarization states for specific voltages are provided in Supplementary Fig. S5 (Supporting Information), measured through a polarimeter (PAX1000VIS).

As a proof of concept, we integrate the LC cell with our metalens and measure the *xz-*plane intensity distributions at voltage settings corresponding to RCP (0.87 V) and LCP (1.03 V), which we refer to as mode 1 (M_1_) and mode 2 (M_2_), respectively (Fig. 3f). The customized measurement setup is detailed in Supplementary Fig. S6 (Supporting Information). Since the measurement is conducted in air, we use a metalens with the same discretized phase distribution but optimized for free-space operation (Supplementary Fig. S7, Supporting Information). The normalized intensity distributions at each focal plane and the corresponding Strehl ratios (SRs) are shown in Supplementary Fig. S8. The SRs of both modes are close to unity, indicating that the focal profiles of both modes closely resemble diffraction-limited Gaussian beams with the corresponding NAs, suggesting that each mode can be regarded as a diffraction-limited Gaussian-beam focus at a different depth. Since the focal length increases by a factor of 1.33 when the metalens is placed underwater, we design the focal length difference to be 0.9 mm in water, setting F_1_ = 4.5 mm and F_2_ = 5.2 mm, which correspond to NA = 0.067 and NA = 0.058, respectively.

To characterize the optical contrast between the two focal modes, we gradually increase the voltage applied to the LC cell, transitioning from M_1_ to M_2_ while measuring the focal spot intensity. The optical contrast between the modes reaches 40:1 at F_1_ and 20:1 at F_2_ (Fig. 3g), demonstrating effective on- and off-focus switching between the modes. Detailed focal intensity variations as a function of applied voltage are provided in Supplementary Fig. S9 (Supporting Information).

The imaging capability of the fabricated dual-focus metalens is evaluated using modulation transfer functions (MTFs) derived from the measured point spread functions. The MTFs closely match those of an ideal diffraction-limited metalens (Supplementary Fig. S10, Supporting Information). Finally, to further validate the imaging performance of our fabricated metalens, we capture images of a negative 1951 USAF target, specifically element 5 from groups 5 at 532 nm (Fig. 3h). The active focal mode switching between M_1_ and M_2_, achieved by varying the voltage applied to the LC cell using a function generator (Agilent 33220 A), operates on a millisecond scale (Supplementary Video S1–3, Supporting Information).

### Characterization of metalens-integrated PAM system

**Fig. 4:**
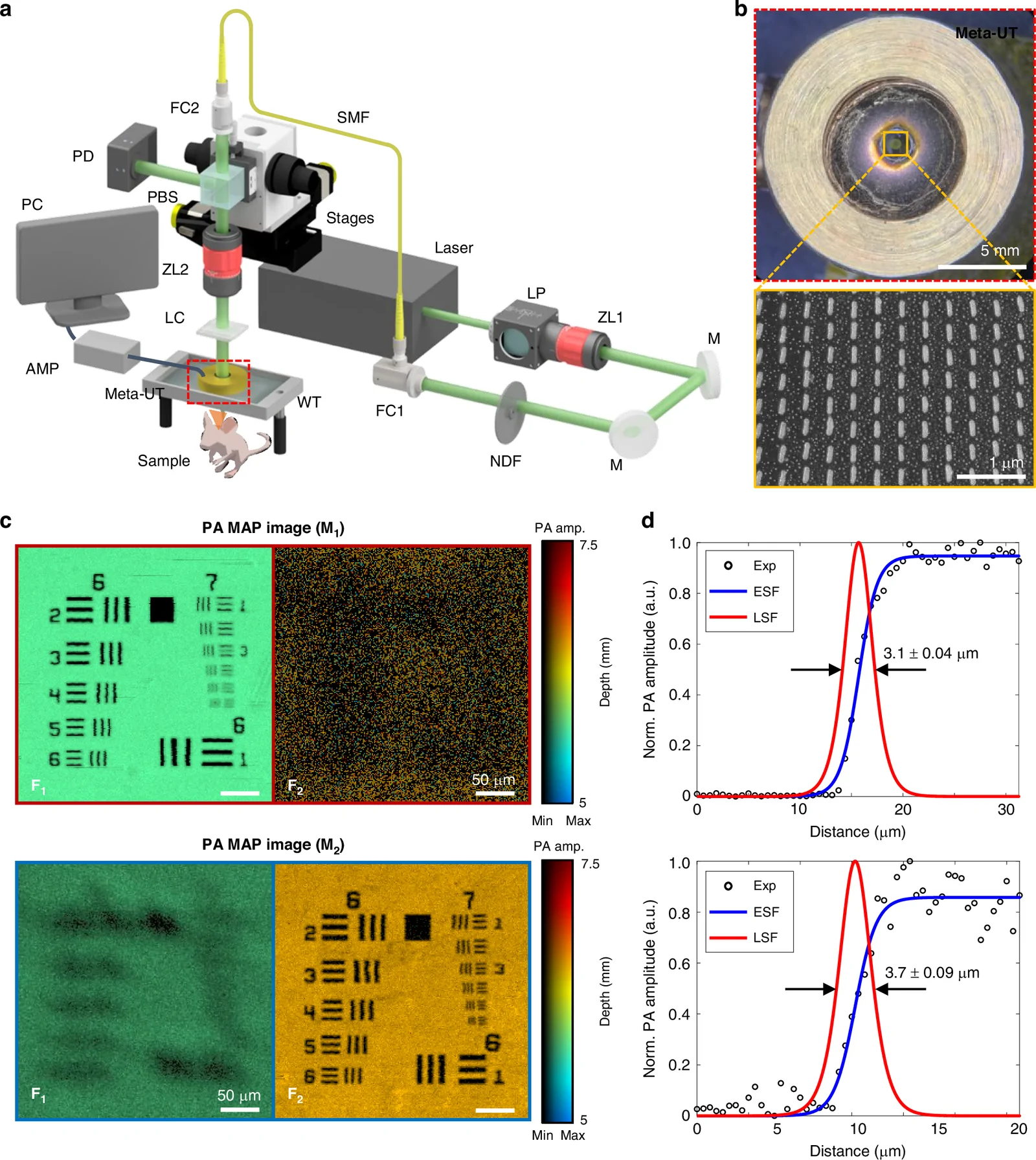
Characterization of dual-focus meta-PAM. **a** Schematic illustration of electrically-modulated dual-focus meta-PAM. **b** Photograph of the meta-UT. **c** PA maximum amplitude projection (MAP) images acquired at different depths (*d*_1_ = 6.05 mm, *d*_2_ = 6.96 mm) using a negative USAF target. Color bars: Hue encodes depth derived from time-of-flight, while brightness represents PA amplitude (Scale bars: 50 μm). **d** Measured lateral resolutions of meta-PAM for both M_1_ and M_2_ modes. FC fiber collimator, SMF single-mode fiber, PD photo diode, PBS polarizing beam splitter, ZL zoom lens, UT ultrasound transducer, WT water tank, LP linear polarization, M mirror, NDF neutral density filter, PC personal computer, ESF edge spread function, LSF line spread function

The integration of the dual-focus metalens into the PAM system, which we term meta-PAM, is illustrated in Fig. 4a (see the details in “Methods”). The dual-focus metalens is mounted on a ring-type ultrasound transducer (meta-UT) to avoid physical interference and ensure coaxial alignment of the optical and acoustic waves (Fig. 4b). Figure 4c shows the imaging performance of the dual-focus meta-PAM system, demonstrating the negative USAF resolution target imaged at different focal modes and depths. The formation of dual focal points for M_1_ and M_2_ in meta-PAM imaging is confirmed at the corresponding imaging depths of F_1,water_ = 6.05 mm and F_2,water_ = 6.96 mm, respectively. The elongation of the focal length is attributed to the change in the surrounding medium from air to water. Furthermore, we measured the laser intensity in the *xy*-plane at multiple depths along the *z*-axis and evaluated the corresponding PA imaging performance (Supplementary Figs. S11, S12, Supporting Information). Each mode provides Gaussian-like single-focus performance and thus functions as an independent single-focus PAM. Switching between M_1_ and M_2_ enables PAM imaging over a substantially extended axial range (~1.2 mm) relative to conventional single-focus PAM.

By calculating rotated nanostructures exhibiting PB phases using a finite difference time domain (FDTD) solver and employing a metalens phase in water using Zemax, we obtain focal length changes from 4.5 to 5.98 mm for M_1_, and 5.2 to 6.91 mm for M_2_ (Supplementary Fig. S13, Supporting Information). The distance between the two optical focal planes in water is 0.93 mm, which closely matches the measured depth difference in the images ($|{z}_{1}-{z}_{2}|=0.91{\rm{mm}}$). Figure 4d depicts the lateral resolution at the focal depth of each mode, quantified as the full width at half maximum (FWHM) of the fitted line spread function derived from the edge spread function. The measured lateral resolutions for M_1_ and M_2_ are 3.1 μm and 3.7 μm, respectively, which are in close agreement with the theoretical resolutions in water (3.0 μm and 3.5 μm, respectively).

### High-resolution PA imaging in dual focal mode

**Fig. 5:**
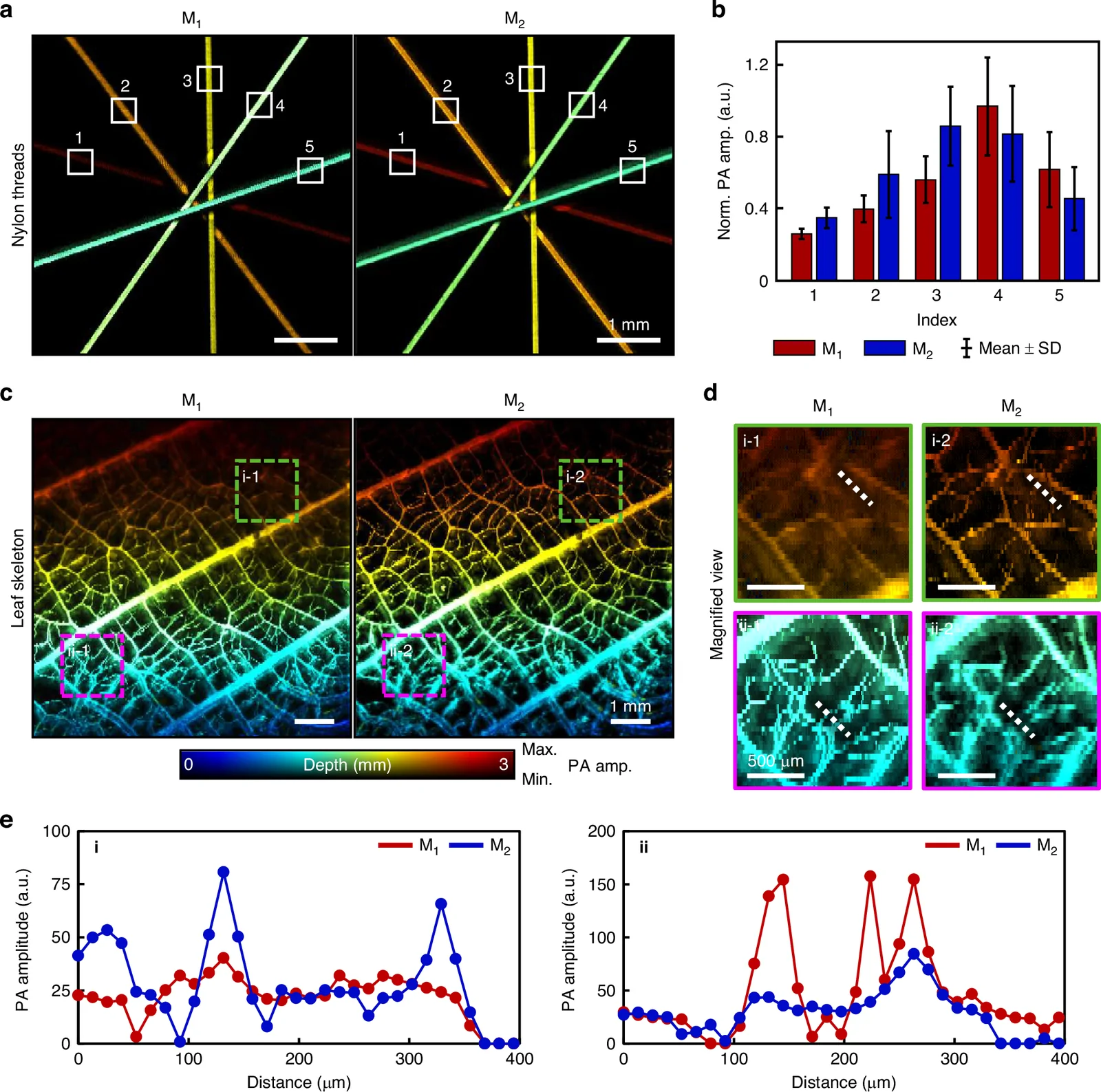
Volumetric PA imaging of nylon threads and a leaf skeleton. **a** Depth-encoded PA MAP image of five axially positioned nylon threads for both M_1_ and M_2_ modes. **b** PA amplitude comparison between M_1_ and M_2_ of each nylon threads. **c** Depth-encoded PA MAP images of a tilted leaf skeleton for both M_1_ and M_2_ modes (Scale bars: 1 mm). **d** Zoomed images of each mode, illustrating the resolution differences between each mode at different axial positions (Scale bars: 500 μm). **e** PA amplitude and resolution comparison for i, and ii (white dotted line) in (**d**)

To demonstrate the axial focal shift capability of the dual-focus meta-PAM system, we image intersecting 30 μm-thick black nylon threads positioned at five discrete heights (Fig. 5a) (Supplementary Fig. S14, Supporting Information). In M_1_, thread 5 is in sharp focus and exhibits the highest contrast, whereas thread 1, located at a deeper axial position, appears blurred. On the other hand, in M_2_, the focal plane shifts axially, bringing thread 2 into focus, with threads in deeper positions showing higher contrast. The quantitative comparisons at each point, indicated by the white boxes in Fig. 5a, are shown in Fig. 5b. Each mode has different image contrast depending on the depth, implying that our meta-PAM system effectively varies the axial PA focal point, demonstrating an electrically tunable dual-focus PAM.

To further investigate the performance of dual-focus meta-PAM for imaging objects with continuous elevation changes, we image a sloped leaf skeleton (Fig. 5c). The imaging results reveal depth-dependent variations in image quality across the two modes. As highlighted in the magnified view (Fig. 5d), region ‘i’ exhibits superior resolution and contrast in M_2_, while region ‘ii’ appears sharper with higher contrast in M_1_. Figure 5e compares PA line profiles extracted from the white dotted lines in Fig. 5d. The in-focus images clearly resolve finer structures and exhibit larger amplitude signals compared to the out-of-focus images, confirming the depth-selective imaging capability of dual-focus meta-PAM. These results validate the effectiveness of dual-focus meta-PAM in enhancing resolution and contrast at wider axial depths, offering a powerful tool for depth-resolved PA imaging. Additionally, we have examined the performance of dual-focus meta-PAM under the scattering condition (Supplementary Fig. S15, Supporting Information). A scattering phantom consisting of water, gelatin, and intralipid was constructed to have an effective scattering coefficient (*μ*_eff_) of 0.9 cm^−1^, and black threads were planted at 0.5 mm depth intervals. As the focal mode switched from M_1_ to M_2_, the contrasts for deeper threads are improved. In particular, the resolution in the thread at a depth of about 1 mm deeper (from #2 to #4) is also improved, confirming that the focus shifted by the metalens.

### In vivo microvascular imaging of small animal

To demonstrate the advantages of the dual-focus meta-PAM system, we perform in vivo imaging of a corneal burn-injured rat eye model and compare the results with a normal rat eye. Due to the inherent curvature of the rat eye, conventional PA imaging with a single optical focus struggles to capture blood vessels located at the periphery. Additionally, corneal neovascularization (CNV) induced by chemical burns occurs within the cornea layer, introducing height variations that further complicate imaging challenges. The dual-focus capability of the meta-PAM system effectively addresses these depth-dependent limitations (Supplementary Fig. S16, Supporting Information), enabling the acquisition of high-resolution PA images across a broader field of view.

**Fig. 6:**
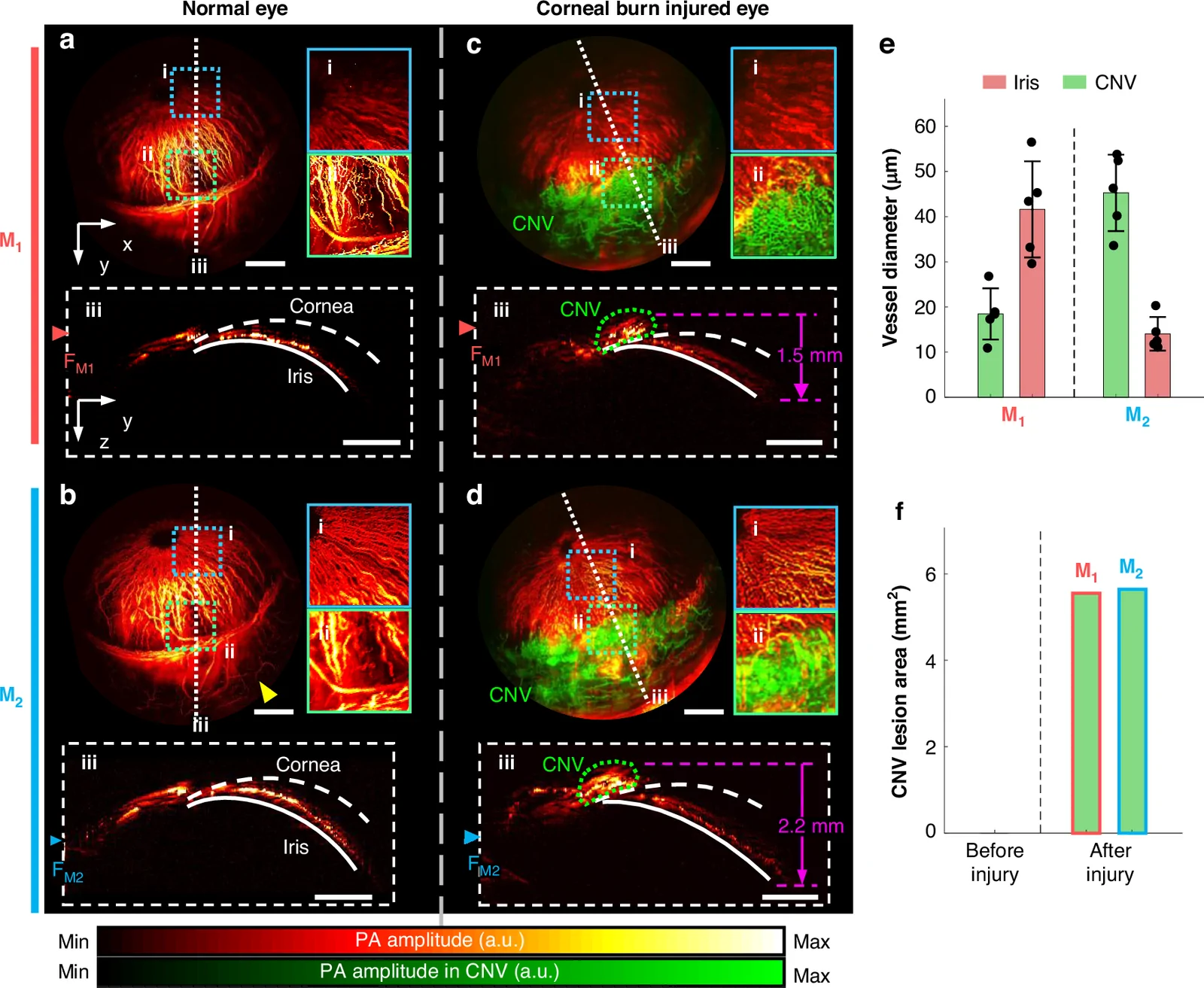
In vivo label-free PA imaging of rat eyes. PA imaging of a normal rat eye with M_1_ (**a**) and M_2_ (**b**). PA imaging of a corneal-burn injured rat eye with M_1_ (**c**) and M_2_ (**d**). (i): Blood vessels located at the periphery of the iris. (ii): Root vessel at the iris root. (iii): Cross-sectional image of the white line showing effective resolution difference. **e** Quantitative comparison of vessel diameters measured from the iris and CNV regions under M_1_ and M_2_. **f** Quantification of CNV lesion area before and after chemical burn injury (Scale bars: 1 mm). CNV: corneal neovascularization

Figure 6a presents an in vivo, label-free PA maximum amplitude projection (MAP) image of a normal rat eye acquired using M_1_ (short focus). The blood vessels within the iris and choroid are clearly visualized. However, as shown in Fig. 6a(i), blood vessels located at the periphery of the iris appear blurred due to their deeper positioning along the *z*-axis. In contrast, the root vessels and adjacent microvasculature, which are at shallower depths, exhibit significantly finer resolution (Fig. 6a(ii)). The cross-sectional image extracted along the white line (Fig. 6a(iii)) further confirms that M_1_ effectively captures blood vessels with high resolution at its focal depth, as indicated by the red arrow.

Figure 6b displays a PA MAP image acquired using M_2_ (long focus). Compared to M_1_, the increased focal depth enables visualization of deeper structures over a wider field of view. Notably, the posterior ciliary vessel, indicated by the yellow arrow, is clearly observed. Additionally, the deeper pupil-associated vasculature, which appeared blurred in M_1_, is now well resolved (Fig. 6b(i)), whereas the root vessel region appears less distinct (Fig. 6b(ii)). The cross-sectional image (Fig. 6b(iii)) further confirms that M_2_ facilitates the visualization of densely distributed blood vessels at greater depths compared to M_1_.

Figure 6c presents a label-free PA MAP image obtained 14 days after inducing an alkali burn in the rat cornea. The newly formed CNV, originating from the root vessel, is mapped in green. Similar to Fig. 6a(i), blood vessels at deeper depths appear blurred at M_1_ (Fig. 6c(i)), while the CNV is distinctly visualized (Fig. 6c(ii)). The cross-sectional image (Fig. 6c(iii)) further illustrates the depth distribution of the newly formed CNV, confirming the presence of neovascular structures that were absent in the normal eye. In addition, PA signals could be detected from iris vessels located as deep as ~1.5 mm beneath the CNV layer. Figure 6d presents the PA MAP image acquired using M_2_ in the CNV rat model. The iris vasculature near the pupil is sharply resolved (Fig. 6d(i)), while the CNV region appears blurred due to its shallow positioning, resulting in a loss of resolution (Fig. 6d(ii)). The cross-sectional image (Fig. 6d(iii)) demonstrates that, in contrast to Fig. 6c(iii), M_2_ provides high-resolution PA signals capturing deeper iris vasculature (~2.2 mm). This corresponds to a 0.7 mm extension in detectable depth compared to the M_1_ focal condition. These findings confirm that while chemical burns trigger CNV formation, the iris vasculature remains completely unaffected, indicating that pathological neovascularization is strictly confined to the cornea.

In addition to qualitative visualization, quantitative analysis was performed to evaluate the performance of the meta-PAM system under in vivo imaging experiments. As shown in Fig. 6e, vessel diameters were quantified from both the iris and CNV regions based on the FWHM of the PA amplitude line-profile (Supplementary Fig. S17, Supporting Information). Under the M_1_ mode, where the optical focus matches the corneal surface, CNV vessels were measured with an average diameter of ~18 μm. By comparison, iris vessels, positioned at a deeper location and therefore evaluated outside the focal zone, exhibited a significantly larger apparent mean diameter of about 42 μm due to defocusing. When the focus was shifted deeper under the M_2_ focal mode, iris vessels were more accurately quantified, yielding an average diameter of ~16 μm. Conversely, CNV vessels, imaged in a defocused condition, exhibited an average diameter of ~45 μm.

Beyond vessel morphology, the pathological extent of CNV was evaluated by quantifying the lesion area before and after chemical burn injury (Fig. 6f). Prior to injury, no CNV was detectable, whereas after injury, the lesion area was quantified as 5.55 mm^2^ and 5.65 mm^2^ under the M_1_ and M_2_ modes, respectively (Supplementary Fig. S18, Supporting Information). Despite focal depth variation affecting local resolution, the CNV lesion area was measured to be nearly identical between modes, indicating that metalens-based dual-focus imaging enables reliable lesion-scale quantification while preserving pathological interpretability across imaging depths. Notably, the vessel diameters and CNV lesion areas measured in the two modes agree closely, indicating that anterior-segment birefringence does not have a significant impact on polarization-dependent artifacts in our meta-PAM measurements.

## Discussion

In this study, we develop an electrically tunable dual-focus meta-PAM system capable of switching imaging modes (532 nm) between two diffraction-limited optical foci, separated by 0.9 mm in the axial direction. The polarization-dependent dual-focus metalens is mounted on the ring-type UT, eliminating the need for additional alignment. Focal mode switching is achieved solely by adjusting the applied voltage on the LC cell on a millisecond timescale, which functions as a QWP, eliminating any mechanical movement of optical components.

Using the dual-focus meta-PAM system, we successfully image nylon threads at different axial positions and sloped leaf skeletons, demonstrating enhanced resolution and image contrast across a broader axial depth range. Furthermore, in vivo imaging of a corneal burn-injured rat eye model reveals that pathological neovascularization remains strictly confined to the cornea, enabled by focal mode switching. Notably, the 0.9-mm focal separation between the two focal planes spans the corneal thickness (typically 0.5–0.6 mm), meaning that clinically relevant corneal layers are imaged within the high-resolution regime. These findings highlight the precision of dual-focus meta-PAM in accurately resolving depth-specific vascular changes, demonstrating its potential as a powerful tool for investigating pathological conditions that require high spatial resolution across an extended depth range. Unlike conventional OR-PAM, which maintains high image quality only near a single focal plane, our dual-focus metalens resolves this intrinsic depth-dependency limitation by enabling high-efficiency focusing at two distinct corneal depths. Therefore, the metalens integration is not simply a focusing modification but an essential optical solution that allows PAM to reliably visualize multilayer vasculature. While SLM-based approaches can provide dynamic wavefront shaping, our metalens-based dual-focus design offers a distinct advantage by employing subwavelength nanostructures that suppress higher-order diffraction, thereby achieving high focusing efficiency and generating stronger PA signals^40^.

Compared to DOF extension methods that modify the illumination beam, such as Bessel beams generated by axicons^15^ and needle beams produced by diffractive optical elements and metalens^41,42^, our approach offers a distinct advantage in practical imaging performance. While Bessel-beam illumination can extend DOF, its strong sidelobes degrade focal intensity and image contrast and necessitate additional complex post-processing. Needle-beam illumination can provide depth-invariant high lateral resolution, but redistributing optical energy over an extended axial range lowers the local focal intensity, leading to reduced PA signal amplitudes and thus inferior SNR compared with our dual-focus approach (Supplementary Fig. S19, Supporting Information). In contrast, our dual-focus metalens enables switchable, diffraction-limited Gaussian focusing, selectively delivering a strong and efficient focus with minimal sidelobes while extending the DOF. This design provides high SNR and robust resolution against scattering and phase distortion across the extended DOF (Supplementary Fig. S20, Supporting Information).

We have applied the meta-PAM with extended DOF to in vivo rat ocular imaging, and the imaging performance can be further improved. First, the imaging speed is a critical parameter for in vivo applications. We have set a frame rate to about 0.42 fps at a 6 mm field of view and 1.25 μm/pixel step size, avoiding a motion error from the stage scanning. The frame rate can be adjusted up to 20 fps with a 100 kHz laser pulse repetition rate using a fast optical scanning mechanism^10,43,44^. High-speed meta-PAM will enable real-time monitoring of dynamic ocular processes. By adding an anterior segment vasculature analysis at multiple time points, the disease severity assessment and the healing process monitoring can be achieved^45^. As imaging speed increases, respiration-induced ocular motion becomes more pronounced; therefore, motion compensation techniques such as frame registration will be needed to preserve image fidelity. Second, during the fabrication phase, the dual-focus metalens is designed to shift focus only in the axial direction. However, switching focal modes changes the optical NA, which may cause inconsistencies between meta-PAM volumetric images. Although no additional calibration is required in switching modes, the subtle mismatch can be corrected with subpixel registration when merging two focal mode images. Seamless merging of focal modes provides DOF-extended PA images without distortion. Designing a multi-focus metalens that considers the optical properties of the eye (structures and refractive indices from the cornea to the lens) will enable the visualization of retinal microvasculatures in the posterior segment^46^. Third, although we have demonstrated meta-PAM in the scattering media, the metalens designed in accordance with the water environment did not fully perform tight focusing and high-SNR (Supplementary Fig. S15, Supporting Information). By designing metalenses that take into account the optical properties of surrounding tissues, meta-PAM can be optimized for various applications, such as skin and brain vasculatures. By contrast, in transparent media such as ocular tissues, meta-PAM effectively achieves high-resolution imaging across a substantial depth range. This capability highlights its strong potential for ophthalmic applications compared to other modalities (Supplementary Fig. S21, Supporting Information)^47–54^.

Although our current design provides adequate performance for corneal microvessel and neovascular imaging, our current implementation can be further improved. First, larger metalenses enabled by scalable manufacturing could increase the NA, allowing higher-resolution OR-PAM. Second, because SiN_*x*_ is dispersive, extending our approach to multispectral PAM will require metasurface designs that correct wavelength-dependent aberrations across distinct excitation wavelengths. Third, the overall optical throughput is mainly limited by incomplete cross-polarization conversion arising from nanofabrication imperfections, whereas material absorption in the optimized SiN_*x*_ film is minimal. Therefore, more precise nanofabrication and further optimization of the SiN_*x*_ deposition process should enhance the optical throughput of meta-PAM.

While this study focuses on applying the tunable focal modes to PA imaging, the underlying concept is broadly applicable. This tunable focal approach can be integrated into various imaging modalities. For instance, integrating the dual-focus metalens into both PAM and OCT could provide complementary contrast, where OCT captures structural details based on optical scattering, while PAM visualizes vascular and functional structures through optical absorption contrast^55^. This combination is particularly useful in ophthalmology and dermatology. In addition, the compact and tunable optics of the meta-PAM system allow seamless integration with two-photon microscopy, providing high-resolution structural and functional imaging at extended imaging depths^56–60^. Furthermore, extending the design to an LC-integrated multi-focal metasurface platform could generate multiple or axially sweeping focal planes with programmable depth weighting. Beyond spatial focusing control, dispersion- and polarization-engineered metasurfaces could also be exploited to realize multispectral and polarization-sensitive PAM, enabling spectroscopic separation of endogenous and exogenous chromophores, quantitative functional readouts such as hemoglobin oxygen saturation^61–66^, and polarization-resolved contrast for probing molecular orientation and structural anisotropy^67–69^. With its multimodal adaptability, meta-PAM presents a versatile platform for advanced biomedical imaging applications, improving diagnostic accuracy and expanding our insights into complex biological systems.

## Materials and methods

### Numerical simulations

Numerical simulations were conducted using the commercially available finite element method (FEM) solver COMSOL Multiphysics 6.1. The simulations of the electric field were performed using periodic boundary conditions in the *x*-and *y*-directions and perfect boundary conditions in the *z-*direction.

### Measurement of refractive index

The refractive index of the SiN_*x*_ film was measured using ellipsometry. The measured amplitude ratio and phase difference between the s and p components were fitted using the Cauchy dispersion model.

### Fabrication of metalens

The metalens was fabricated on a glass substrate, where a 700 or 1000 nm thick layer of SiN_*x*_ film was deposited on the substrate using plasma enhanced chemical deposition with SiH_4_/N_2_ flow ratios of 1.52. The meta-atom structures were exposed onto a positive tone photoresist (Microchem, 950 PMMA A2) using a general electron beam lithography process (ELIONIX, ELS-7800, 80 kV acceleration voltage and 100 pA beam current). The exposed photoresist patterns were developed in methyl isobutyl ketone/Isopropyl alcohol (1:3) solution. Then, a 70 nm thick chromium (Cr) layer was deposited using electron beam evaporation (KVT, KVE-ENS4004), followed by a lift-off process using acetone. The Cr was used as an etch mask for the SiN_*x*_ in a dry etching process (DMS, silicon/metal hybrid etcher). The remaining Cr etch mask was removed using Cr etchant (CR-7).

### LC cell fabrication

To function as a polarization modulator, the LC cell was fabricated using glass substrates coated with indium tin oxide (ITO) and polyimide alignment layers (Nissan Chemical Korea). The polyimide was applied via spin-coating at 1000 rpm for 10 s, followed by 2500 rpm for 30 s. After spin-coating, the substrates were baked at 230 °C for one hour. The polyimide alignment layer was then mechanically rubbed to induce uniform LC orientation. Two such ITO-coated glass substrates were assembled with a 10 µm gap, maintained using a combination of glass spacers and UV-curable adhesive (NOA 65, Norland Products Inc.).

### Fabrication and characterization of UT

The ring-shaped UT was fabricated using a polyvinylidene fluoride (PVDF) film (PolyK Technologies, LLC, USA) as the piezoelectric material. The PVDF film had a thickness of 18 μm and was cut into a 8 mm diameter disk. Both surfaces of the PVDF film were coated with chromium (50 Å) and gold (100 Å) using a sputtering system (JUWON TECH, Republic of Korea) to form conductive electrodes. The PVDF film was centrally positioned within the housing, and its backside was electrically connected using a small amount of conductive silver epoxy (E-solder 3022, Von Roll Isola, USA) to attach a copper wire for signal transmission. To secure the PVDF film within the housing, a non-conductive epoxy backing layer (Epotek 301, Epoxy Technology, Inc., USA) was applied and cured to a thickness of ~3 mm. The backside electrode of the PVDF film was electrically connected to the pin of a connector, which was then affixed to the housing using conductive epoxy. To achieve acoustic beam focusing, a bearing ball with a diameter of 12 mm was pressed against the front surface of the PVDF film using a heating press, creating a controlled physical curvature. The front surface of the PVDF film was then coated with chromium and gold to establish an electrical ground connection with the housing. For the integration of the metalens, a concentric hole with a diameter of 1.8 mm was precisely drilled at the center of the PVDF film. Finally, a 5 μm-thick parylene layer was deposited onto the front surface to serve as an acoustic matching layer. An image of the fabricated ring-shaped UT is shown in Supplementary Fig. S22 (Supporting Information). To evaluate the performance of the fabricated UT, a pulse-echo test was conducted. The UT was connected to a pulser-receiver (DPR500, JSR Corp., Japan), and a slide glass target was submerged in water for acoustic characterization. The resulting measurements determined a center frequency of 25 MHz and a −6 dB bandwidth of 106%.

Coaxial alignment between the optical and acoustic foci was ensured by the assembly of the meta-UT unit. The dual-focus metalens was first centered on the aperture of the ring-type UT under a microscope. The aligned meta-UT was then rigidly fixed to a supporting rod and installed onto the system without further mechanical adjustment.

### Dual-focus meta-PAM system

We used a nanosecond Q-switched laser (Onda 532, Bright Solutions, Italy) with a center wavelength of 532 nm as a light source for the dual-focus meta-PAM system. The emitted laser beam was initially set using a variable polarizing beam splitter (VA5-532/M, Thorlabs) and zoom lens (GBE02-A, Thorlabs). The power of the collimated beam was attenuated via a variable neutral density filter (NDC-50C-4M, Thorlabs) before entering a reflective fiber collimator (RC04FC-F01, Thorlabs). A single-mode fiber (P1-460B-FC-1, Thorlabs) with a fiber collimator (F240FC-532, Thorlabs) delivers the laser beam, and a polarizing beam splitter (CCM1-PBS251/M, Thorlabs) fixed the initial polarization state. An LC cell was integrated to control the polarization state incident on the metalens. Meta-UT was coaxially assembled with metalens and a customized ring-type UT. The PA signals were amplified using a low-noise 50 dB amplifier (PE15A1013, Pasternack) and collected by a data acquisition (DAQ) board (NI PCIe-6320, National Instruments) and a 12-bit digitizer (ATS9350, Alazartech) with a sampling rate of 500 MS/s. The operating program was built using LabVIEW software (LabVIEW 2017, National Instruments) and constructed 3D data by raster scanning with *xy* motorized stages (L-509.10SD00, Physik Instrumente). During imaging, a photodetector (DET10A/M, Thorlabs) monitored the laser power.

### Animal imaging experiment

All animal experimental procedures were conducted in accordance with the laboratory animal protocols approved by the Institutional Animal Care and Use Committee (IACUC) of Pohang University of Science and Technology (POSTECH-2024-0025-C3). For the in vivo imaging experiments, 4-week-old Sprague-Dawley rats weighing ~150 g were used. Anesthesia was induced using an anesthetic gas system (VIP 3000 Veterinary Vaporizer, Midmark, USA), which supplied 3–5% isoflurane in oxygen at a flow rate of 1.5 L/min. The anesthetized rat was positioned on the imaging bed, and PA imaging was performed on the normal rat eye. The air gap between the water tank and the eye was filled with acoustic gel to match the acoustic impedance. The irradiated laser fluence was adjusted, ensuring no damage to the eye. To induce CNV, a chemical burn was applied to the rat’s eye. Specifically, a filter paper soaked in a 1 N concentration of NaOH solution was placed on the cornea for 30 s, followed by thorough rinsing with phosphate-buffered saline (PBS). Then, the rats were housed under standard conditions for an appropriate period to allow CNV formation. After 14 days, PA imaging was performed under the same conditions and imaging protocol as the initial experiment. The acquired 3D PA datasets were processed and analyzed using the 3D PHOVIS tool in MATLAB software (R2022a, MathWorks, USA)^70^.

## Supplementary information

Supplementary Information

Supplementary Video 1

Supplementary Video 2

Supplementary Video 3
