# Supplementary material for: Electrically switchable dual-focus metalens for depth-extended in vivo photoacoustic imaging of ophthalmic vascular disease
Source: Light Sci Appl. 2026 Sep 4;15:369. doi: 10.1038/s41377-026-02365-8 (PMC13545080; doi:10.1038/s41377-026-02365-8)
Supplement: Supplementary file 1 [file 41377_2026_2365_MOESM1_ESM.pdf]

Supplementary Information for

Electrically switchable dual-focus metalens for depth-extended in vivo photoacoustic imaging of ophthalmic vascular disease

Hongyoon Kim[1,#], Eunwoo Park[2,#], Jeongwoo Park[3,4,5,6,#], Dong Kyo Oh[1], Jehyeon Shin[7], Hyeonsu Heo[1], Chulhong Kim[1,2,3,8,*], Junsuk Rho[1,3,9,10,11,*]

[1]Department of Mechanical Engineering, Pohang University of Science and Technology (POSTECH), Pohang 37673, Republic of Korea

[2]Department of Convergence IT Engineering, Pohang University of Science and Technology (POSTECH), Pohang 37673, Republic of Korea

[3]Department of Electrical Engineering, Pohang University of Science and Technology (POSTECH), Pohang 37673, Republic of Korea

[4]Department of Biomedical Convergence Science and Technology, Kyungpook National University, Daegu 41566, Republic of Korea

[5]Department of Advanced Bioconvergence, Kyungpook National University, Daegu 41566, Republic of Korea

[6]Cell and Matrix Research Institute, Kyungpook National University, Daegu 41944, Republic of Korea

[7]Graduate School of Artificial Intelligence, Pohang University of Science and Technology (POSTECH), Pohang 37673, Republic of Korea

[8]Department of Medical Science and Engineering, Pohang University of Science and Technology (POSTECH), Pohang 37673, Republic of Korea

[9]Department of Chemical Engineering, Pohang University of Science and Technology (POSTECH), Pohang 37673, Republic of Korea

[10]POSCO-POSTECH-RIST Convergence Research Center for Flat Optics and Metaphotonics, Pohang 37673, Republic of Korea

[11]National Institute of Nanomaterials Technology (NINT), Pohang 37673, Republic of Korea

#These authors contributed equally

*Corresponding author. E-mail:

chulhong@postech.edu, jsrho@postech.ac.kr

Supplementary Note 1. Design for independent phase control of incident spin states.

Given that the meta-atom of metalens is anisotropic with subwavelength dimensions, the transmission coefficient can be expressed by a Jones matrix:

$$J(\theta) = R(-\theta)\begin{bmatrix} t_{\mathrm{f}} & 0 \\ 0 & t_{\mathrm{s}} \end{bmatrix} R(\theta) \quad (1)$$

where $R(\theta)$ is the rotation matrix, and t_{f}, and t_{s}, are the complex transmission coefficients along the fast and slow axes, respectively.

When the metalens is illuminated with different spin states ($|\sigma^{+}\rangle$ for RCP and $|\sigma^{-}\rangle$ for LCP), the transmitted electric field can be written as:

$$E_{\mathrm{trans}} = J(\theta) \cdot |\sigma^{\pm}\rangle = \frac{t_{\mathrm{f}} + t_{\mathrm{s}}}{2}|\sigma^{\pm}\rangle + \frac{t_{\mathrm{f}} - t_{\mathrm{s}}}{2} e^{i2\theta}|\sigma^{\mp}\rangle \quad (2)$$

where t_{f} and t_{s} can be expressed as $T_{\mathrm{f}}e^{i\phi_{\mathrm{f}}}$, and $T_{\mathrm{s}}e^{i\phi_{\mathrm{s}}}$, respectively. Here, ϕ_{f} and ϕ_{s} represent the phase retardation along the fast and slow axes.

The first term in Equation 2 corresponds to the co-polarized component, associated with the propagation phase, where phase retardation is controlled by adjusting the meta-atom geometry. The second term represents the cross-polarized component, associated with the Pancharatnam-Beery (PB) phase, where phase modulation is achieved by rotating the meta-atoms. To maximize the PB phase conversion efficiency, the meta-atom should function as an effective half-wave plate, satisfying $|\phi_{\mathrm{f}} - \phi_{\mathrm{s}}| = \pi$ and $|T_{\mathrm{f}}| = |T_{\mathrm{s}}|$.

For independent phase control of the two spin states, the metasurface must satisfy the following equation:

$$J(x,y) \cdot |\sigma^{+}\rangle = e^{i\phi^{-}(x,y)}|\sigma^{-}\rangle,\ J(x,y) \cdot |\sigma^{-}\rangle = e^{i\phi^{+}(x,y)}|\sigma^{+}\rangle \quad (3)$$

where $\phi^{+}(x,y)$ and $\phi^{-}(x,y)$ represent the required phase profiles for the RCP and LCP spin states, respectively. These phases can be written as $\phi^{\pm}(x,y) = \phi_f \pm 2\theta$, where $|\phi_f - \phi_s| = \pi$. By rearranging Equation 3, the Jones matrix $J(x,y)$ can be expressed as

$$J(x,y) = 0.5 \times \begin{bmatrix} e^{i\phi^{+}(x,y)} + e^{i\phi^{-}(x,y)} & ie^{i\phi^{-}(x,y)} - ie^{i\phi^{+}(x,y)} \\ ie^{i\phi^{-}(x,y)} - ie^{i\phi^{+}(x,y)} & -e^{i\phi^{+}(x,y)} - e^{i\phi^{-}(x,y)} \end{bmatrix} \quad (4)$$

therefore, Equation 4 demonstrates that independent phase modulation for both RCP and LCP inputs can be achieved by engineering the phase $\phi^{\pm}(x,y)$ using a combination of propagation and PB phases.

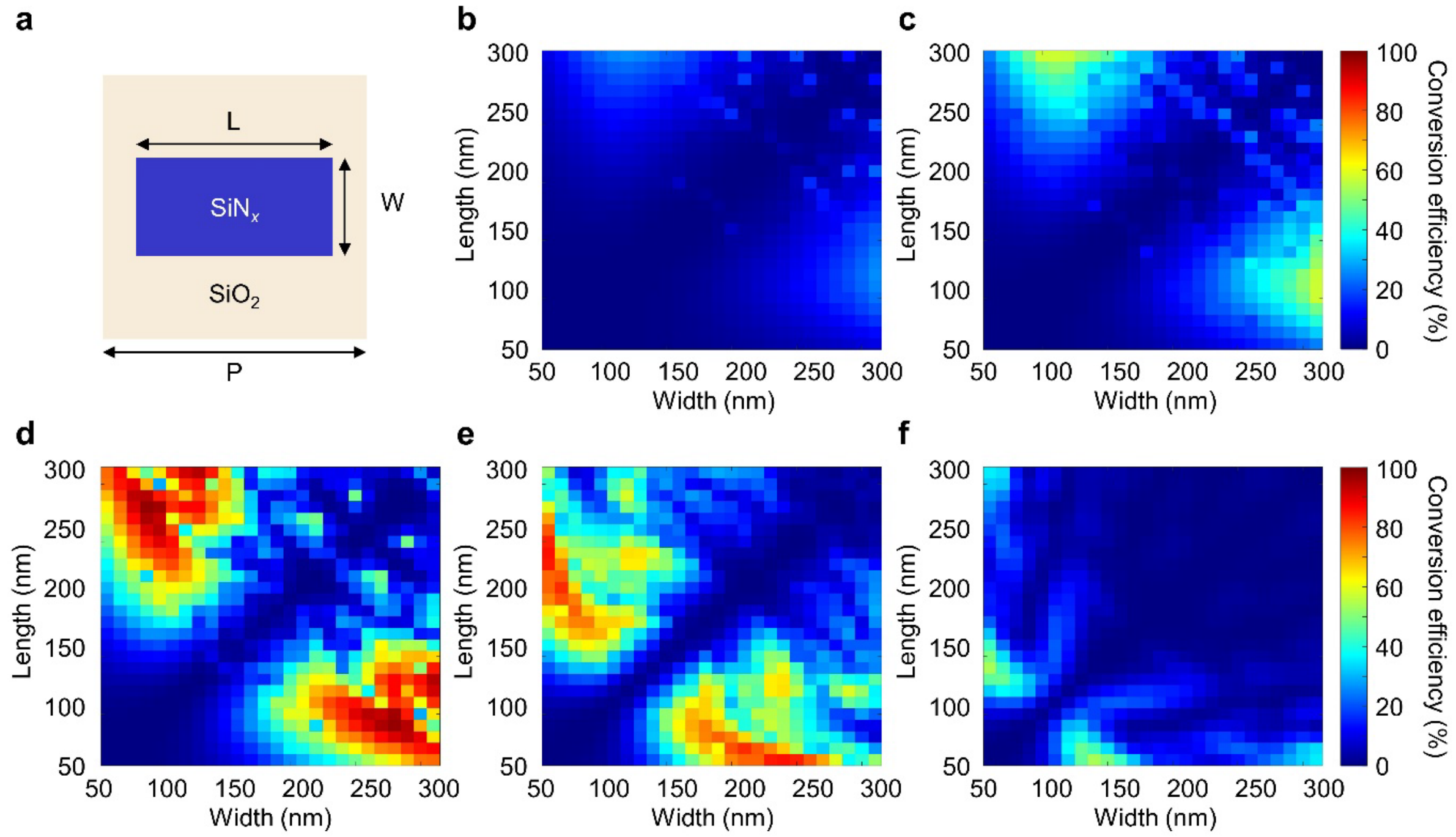

Figure S1. Simulated conversion efficiency of 1000 nm-thick SiN_x meta-atoms with varying SiH_4/N_2 ratios. (a) Schematic of the simulated meta-atom. Conversion efficiencies of SiN_x structures deposited with SiH_4/N_2 ratios of 1.0 (b), 1.2 (c), 1.52 (d), 2.06 (e), and 3.18 (f) are calculated for a 340 nm period on a SiO_2 substrate, while varying the length and width. The surrounding medium is modeled as water (n = 1.33).

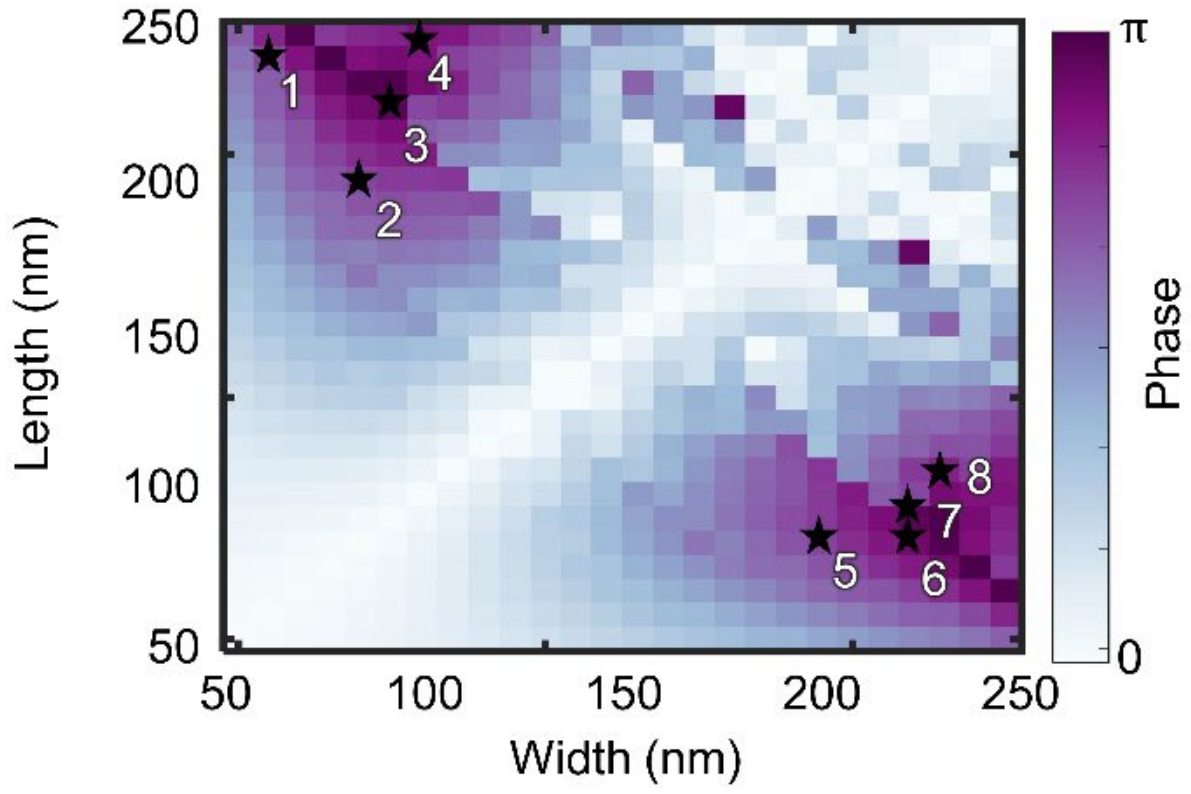

Figure S2. Simulated phase difference between *x*- and *y*-polarized incident light.

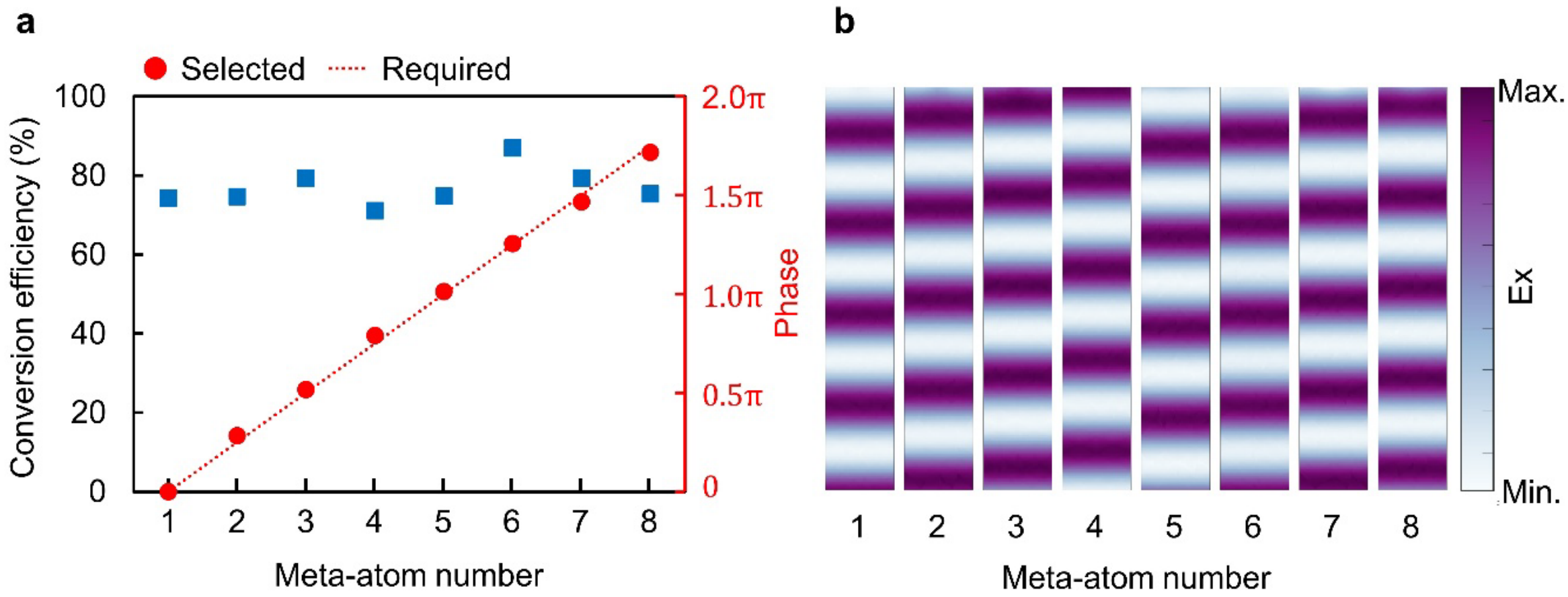

Figure S3. Simulated optical characteristics of the selected meta-atoms under 532 nm wavelength illumination. (a) Calculated conversion efficiency (square) and phase retardation (circle) of the selected meta-atoms. (b) Simulated electric field distribution for different selected meta-atoms under *x*-polarized light.

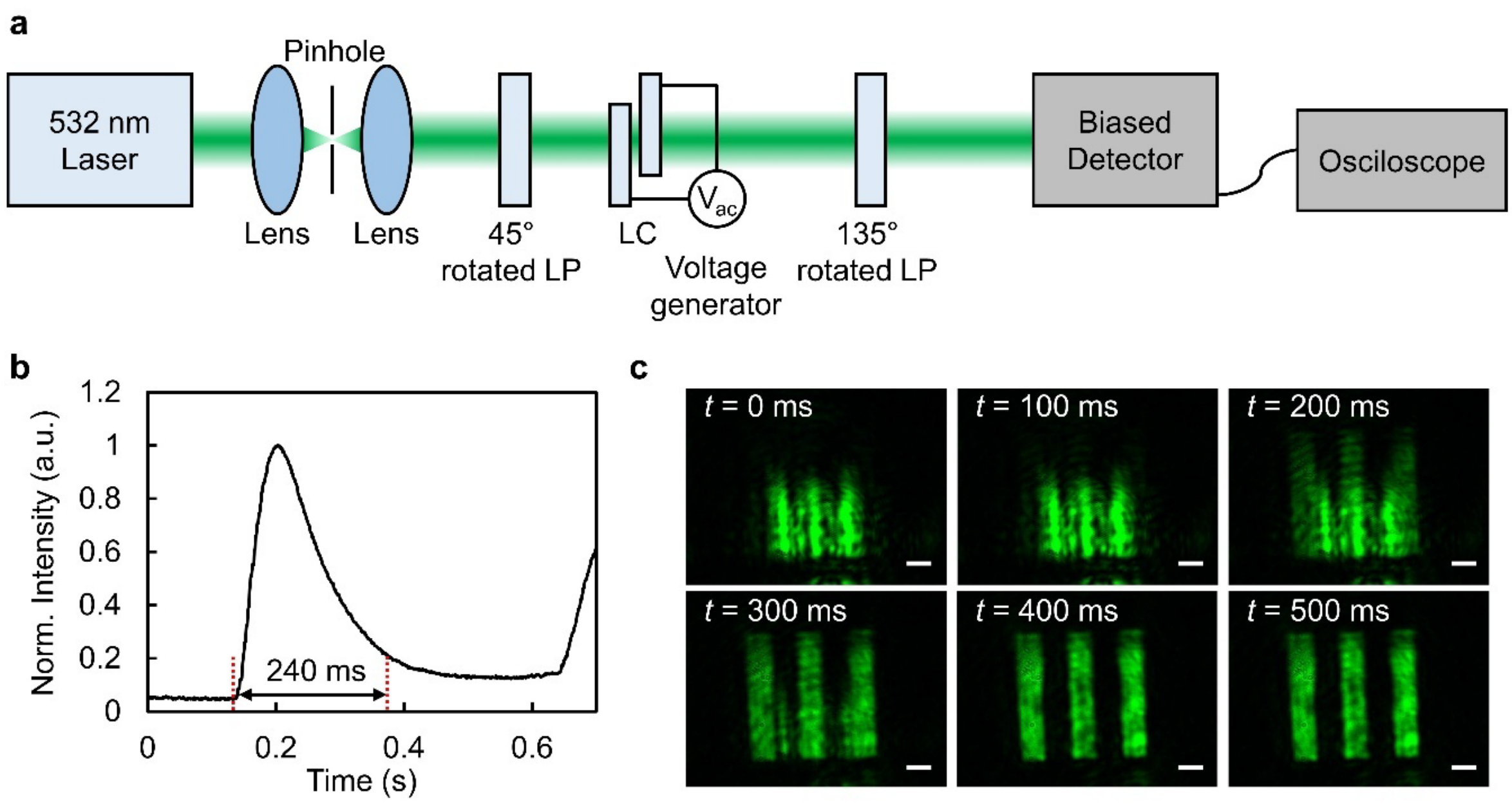

Figure S4. Response time measurement of the LC cell. (a) Schematic of the customized experimental setup used to measure the LC response time. (b) Normalized transmittance of the LC cell as a function of time when a 500 ms-period square-wave voltage is applied. The photodetector monitors the temporal change in transmittance, and the LC response time is defined as the time between the initial rise and the point at which the transmittance reaches 90% decrease of its steady-state value. (c) Time-lapse imaging results of the LC-integrated metalens acquired at 100 ms intervals, showing a similar response time (~240 ms) to that obtained from the transmittance measurement.

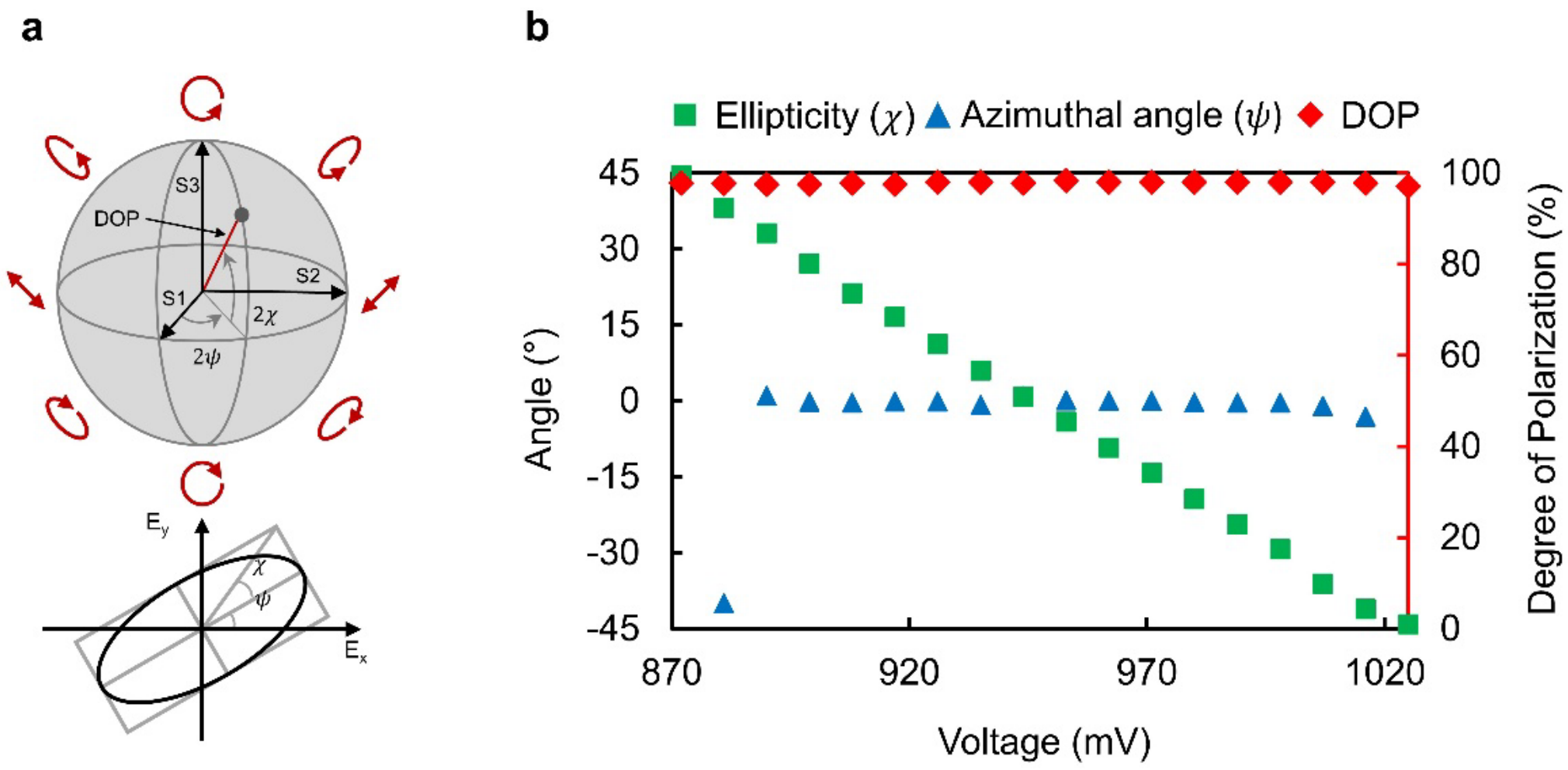

Figure S5. Polarization states as a function of the applied voltage on the LC cell. (a) Polarization states mapped onto the Poincaré sphere. (b) Measured polarization states as the applied voltage varies in the LC cell.

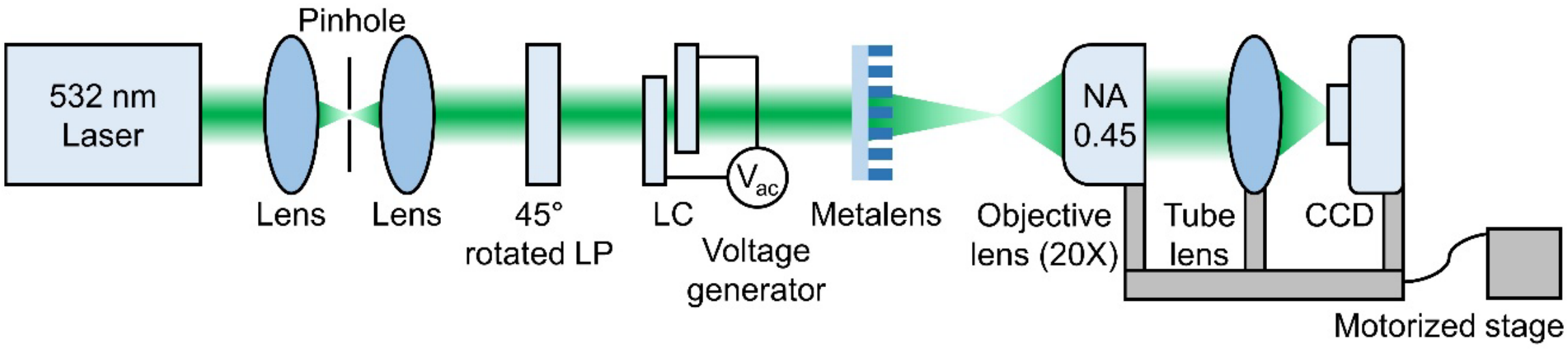

Figure S6. Schematic of the customized optical setup for the characterization of an electrically tunable metalens.

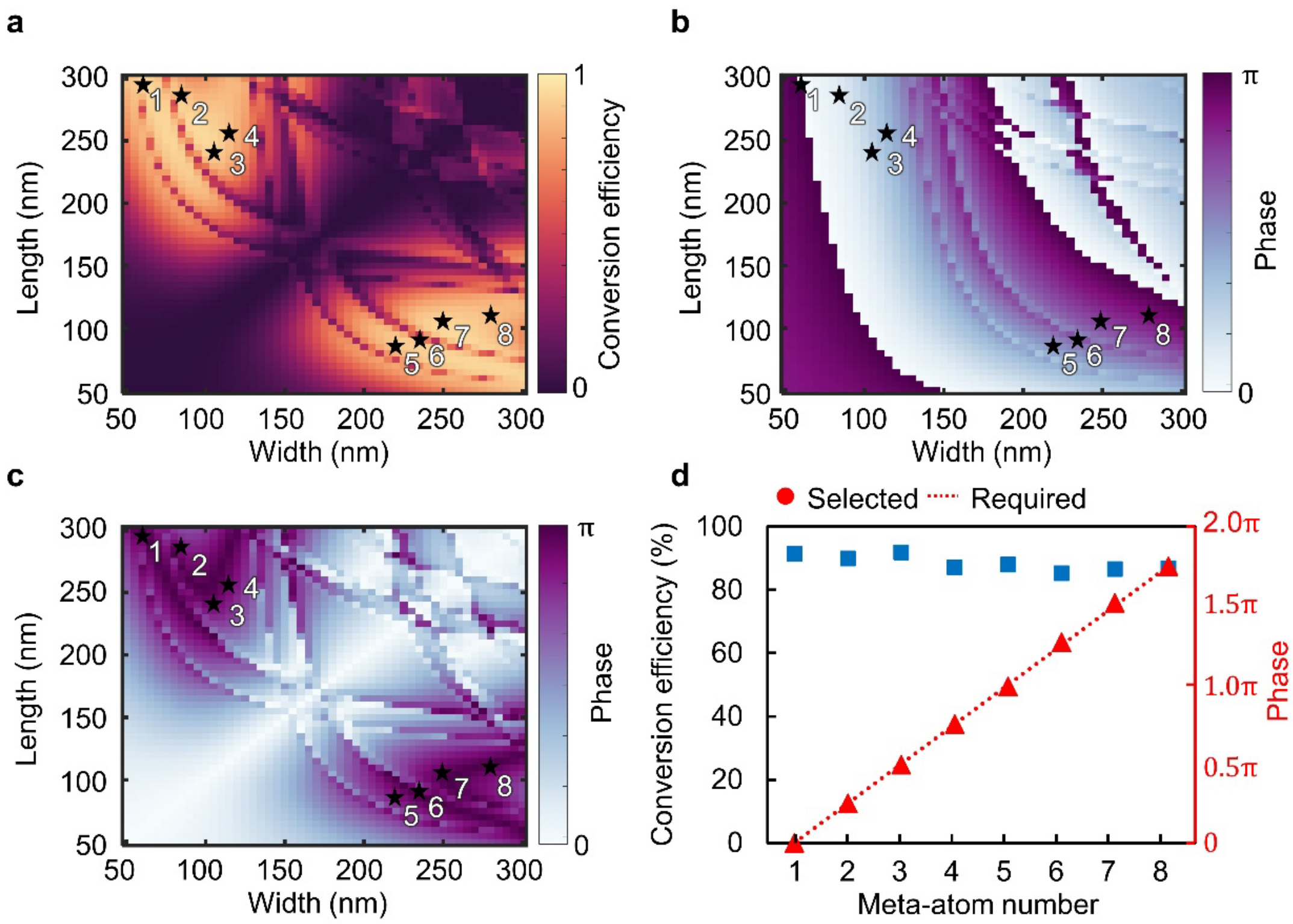

Figure S7. Selected meta-atoms for the metalens with air as the surrounding medium. (a) Simulated conversion efficiency of SiN_x meta-atoms with a height of 700 nm as a function of varying widths and lengths. (b) Simulated phase retardation map. (c) Simulated phase difference map. (d) Simulated conversion efficiency (squares) and phase retardation (circles) of the selected meta-atoms.

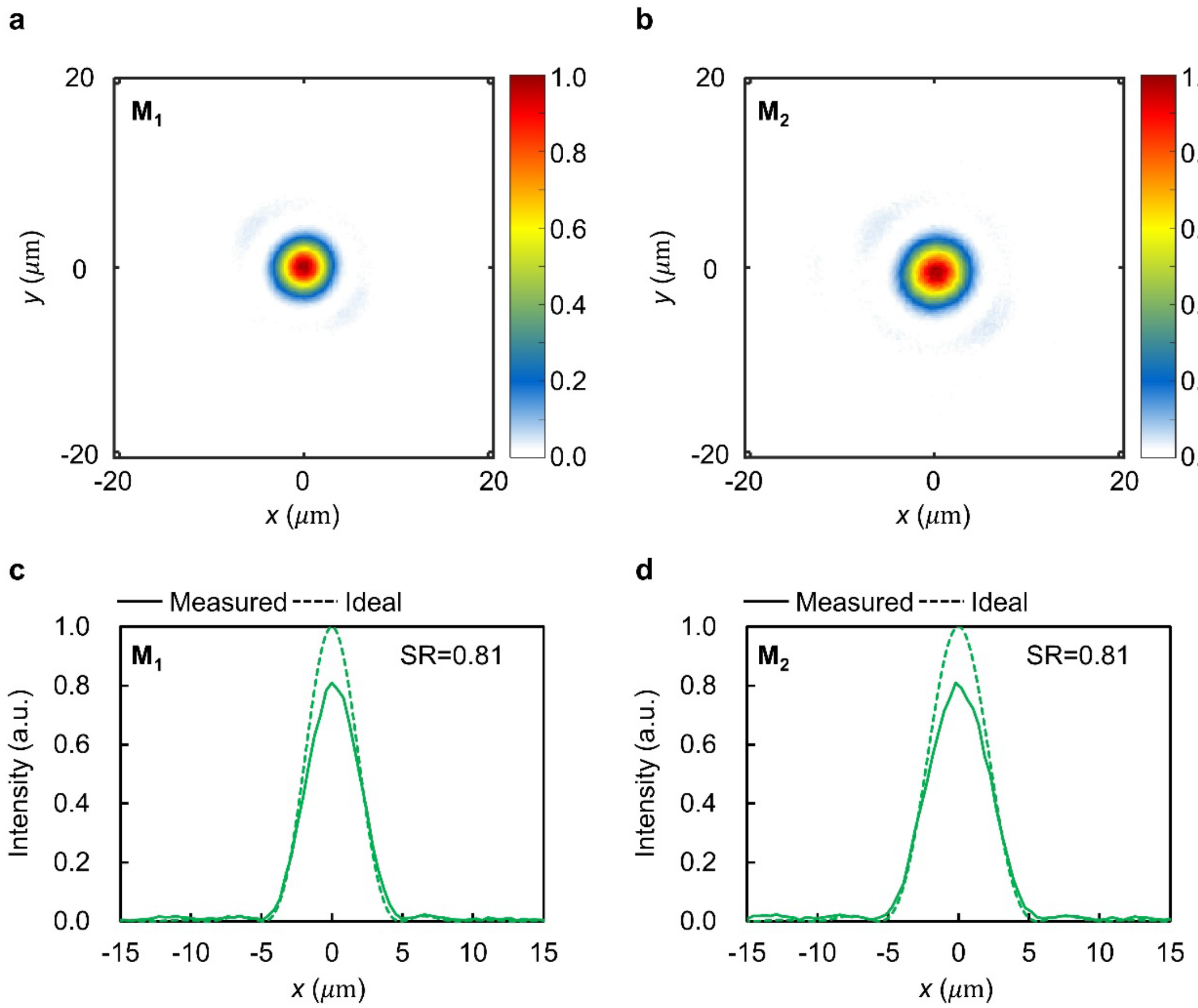

Figure S8. Optical characterization of the fabricated metalens. (a, b) Measured normalized intensity distributions at the focal planes for M_1 (a) and M_2 (b). (c, d) Corresponding focal intensity line profiles, comparing the measured results with the ideal focusing intensity.

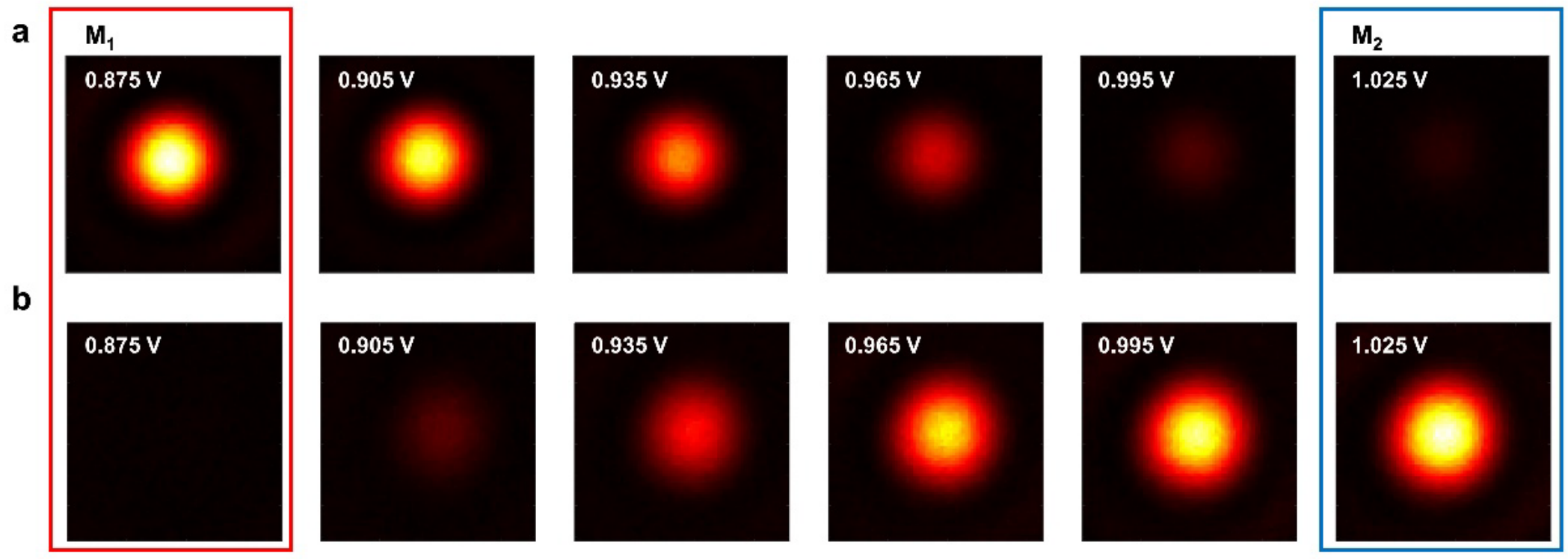

Figure S9. Measured intensity distributions at the focal planes under varying applied voltage on the LC. (a) Intensity distribution variations at F_1= 4.5 mm. (b) Intensity distribution variations at F_2 = 5.2 mm.

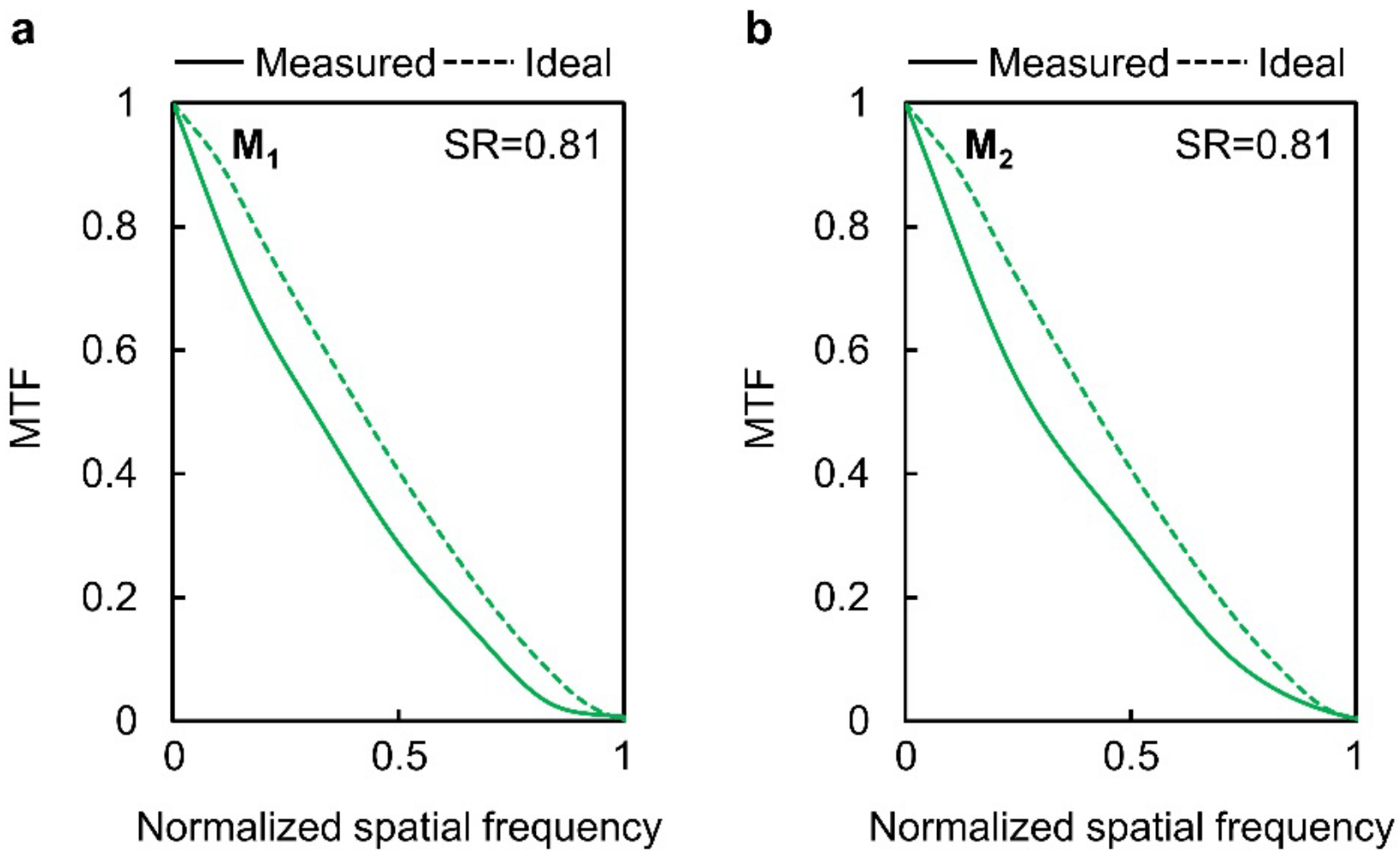

Figure S10. MTF comparison between the ideal imaging system and the fabricated dual-focus metalens. (a) MTF of M_1 (F_1 = 4.5 mm). (b) MTF of M_2 (F_2 = 5.2 mm).

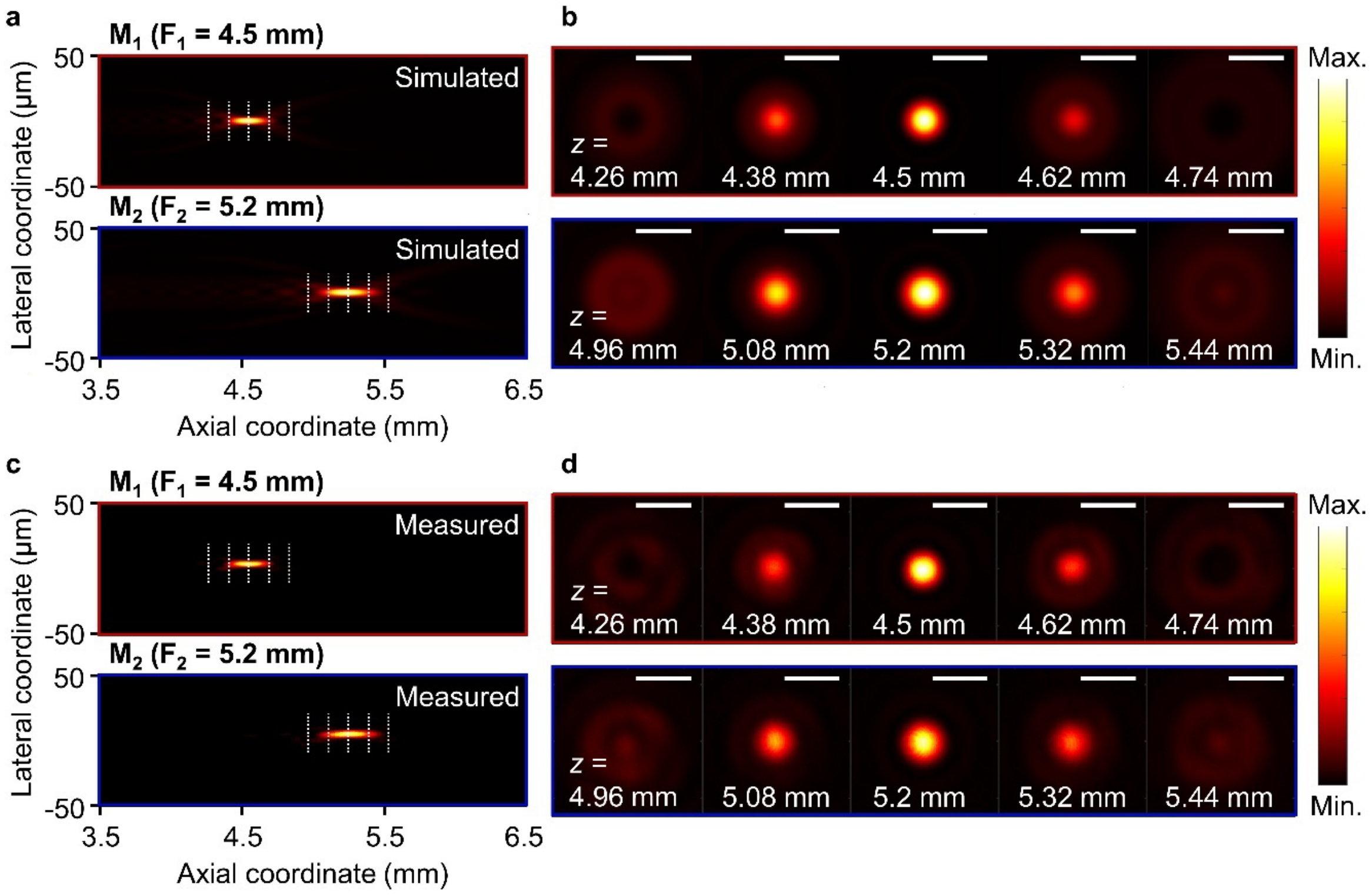

Figure S11. Intensity distribution of the dual-focus metalens. (a) Simulated focal intensity distributions in the *xz*-plane for each mode. Each focus is modeled as a diffraction-limited Gaussian beam at its corresponding focal position using the Rayleigh–Sommerfeld diffraction formula. (b) Simulated focal intensity distributions in the *xy*-plane at different focal depths (*z*) for each mode. (c) Measured focal intensity distributions of the fabricated metalens in the *xz*-plane for each mode. (d) Measured focal intensity distributions in the *xy*-plane at different focal depths (*z*) for each mode (scale bars: 10 μm).

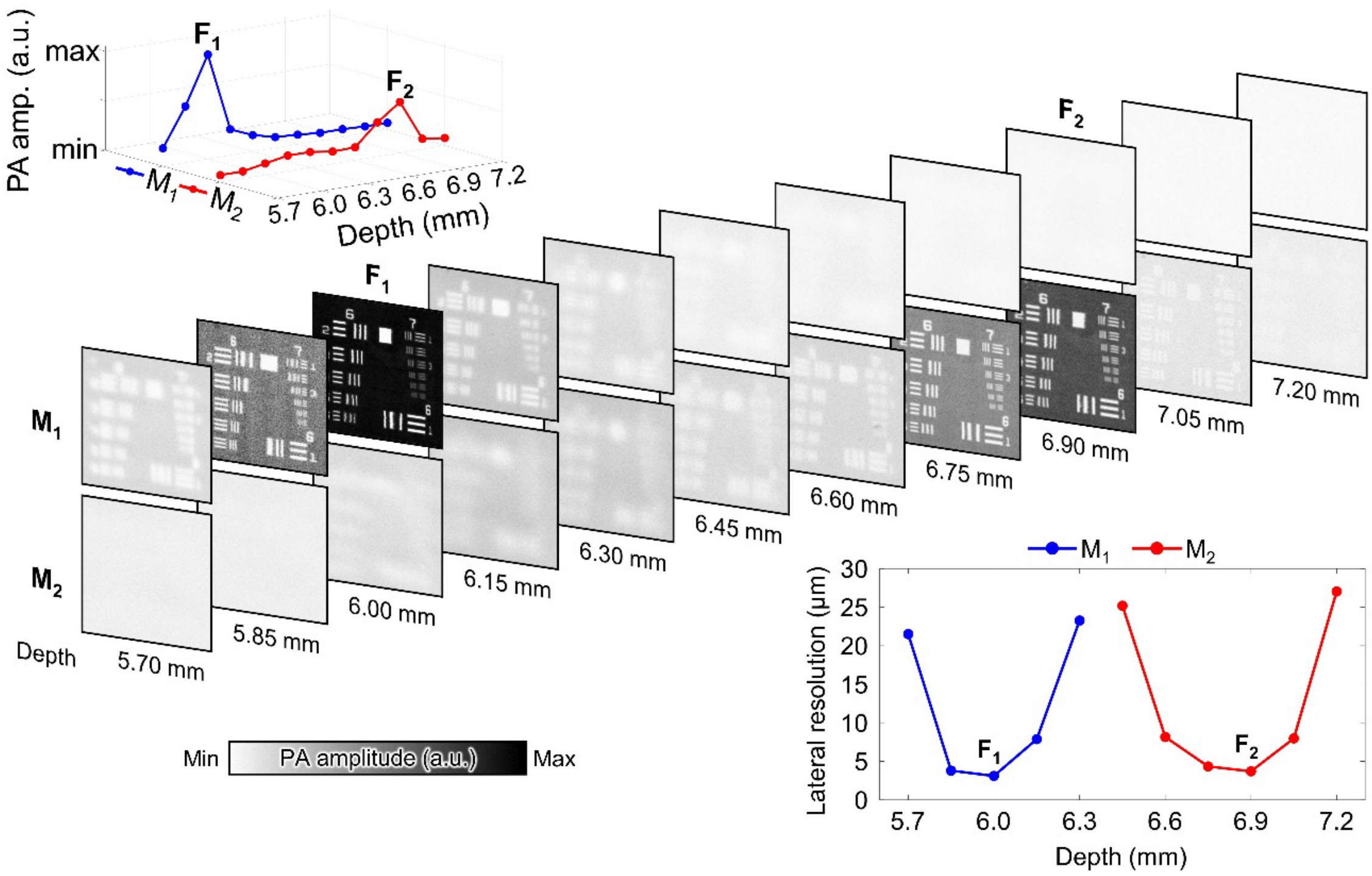

Figure S12. Measured photoacoustic (PA) maximum amplitude projection (MAP) images acquired at multiple depths for M_1 and M_2 modes. Quantified PA amplitudes and measured lateral resolutions are also provided for each depth.

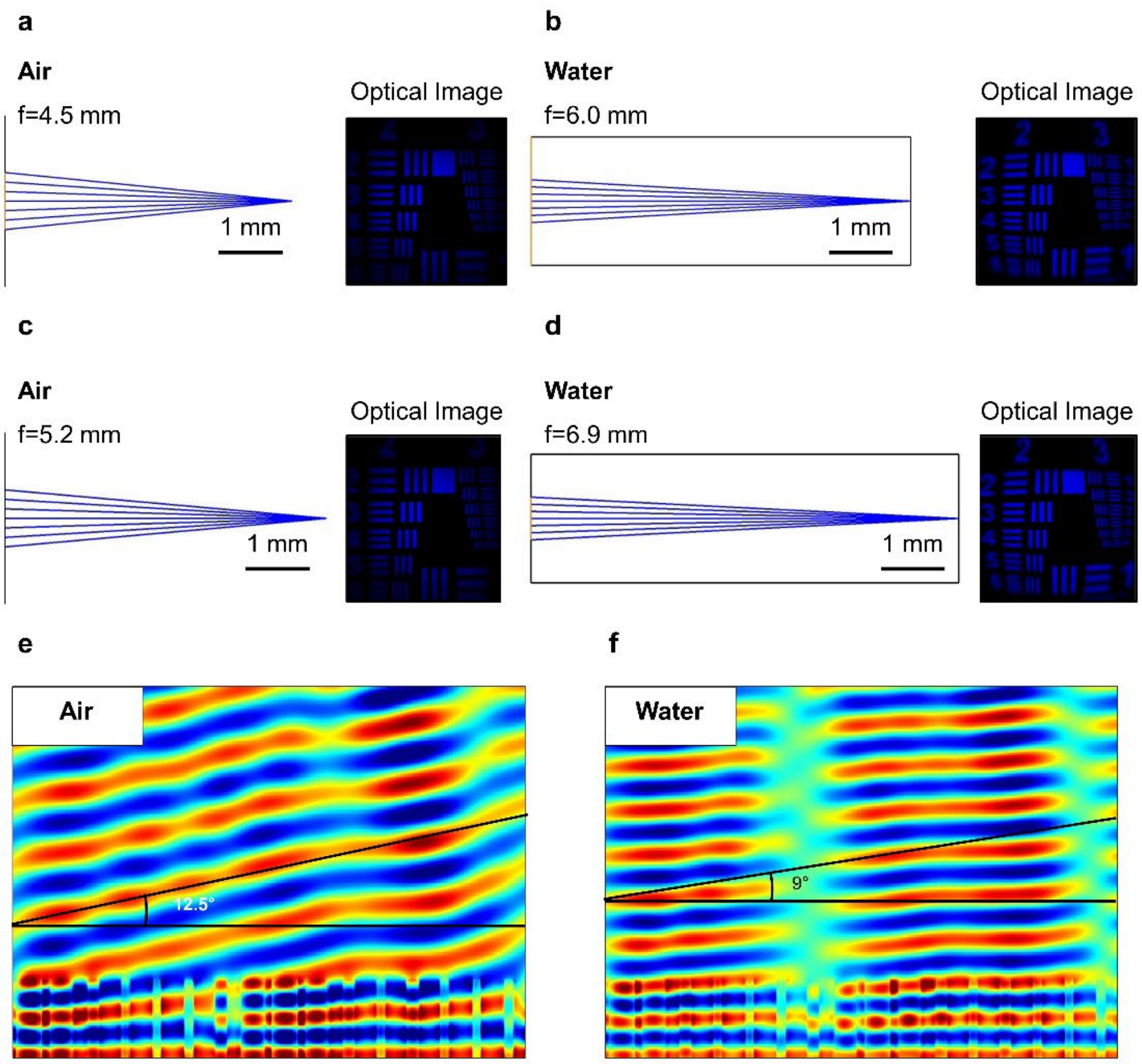

Figure S13. Optical characterization of the metalens focal length in different surrounding media. Simulated focal length and corresponding optical images of the metalens:(a) M_1 in air, (b) M_1 in water, (c) M_2 in air, and (d) M_2 in water. Simulated electric field distributions through PB phased nanostructures with $\pi/4$ phase increments in (a) air and (b) water.

To realize the phase maps of M_1 and M_2 in ZEMAX, each metasurface is modeled as a binary 2 surface, incorporating only even powers of the radial coordinate. To enable this representation, each lens equation is approximated by a Taylor series expansion up to the eighth order for focal lengths of 4.5 mm and 5.2 mm, as described below.

$$\phi(r) \approx -\frac{2\pi}{\lambda}\left(\frac{r^2}{2f} - \frac{r^4}{8f^3} + \frac{r^6}{16f^5} - \frac{5r^8}{128f^7}\right) \quad (1)$$

Simulations are conducted in both air (Figure S11a,c) and water (Figure S11b,d), illustrating the respective ray propagation and phase distributions.

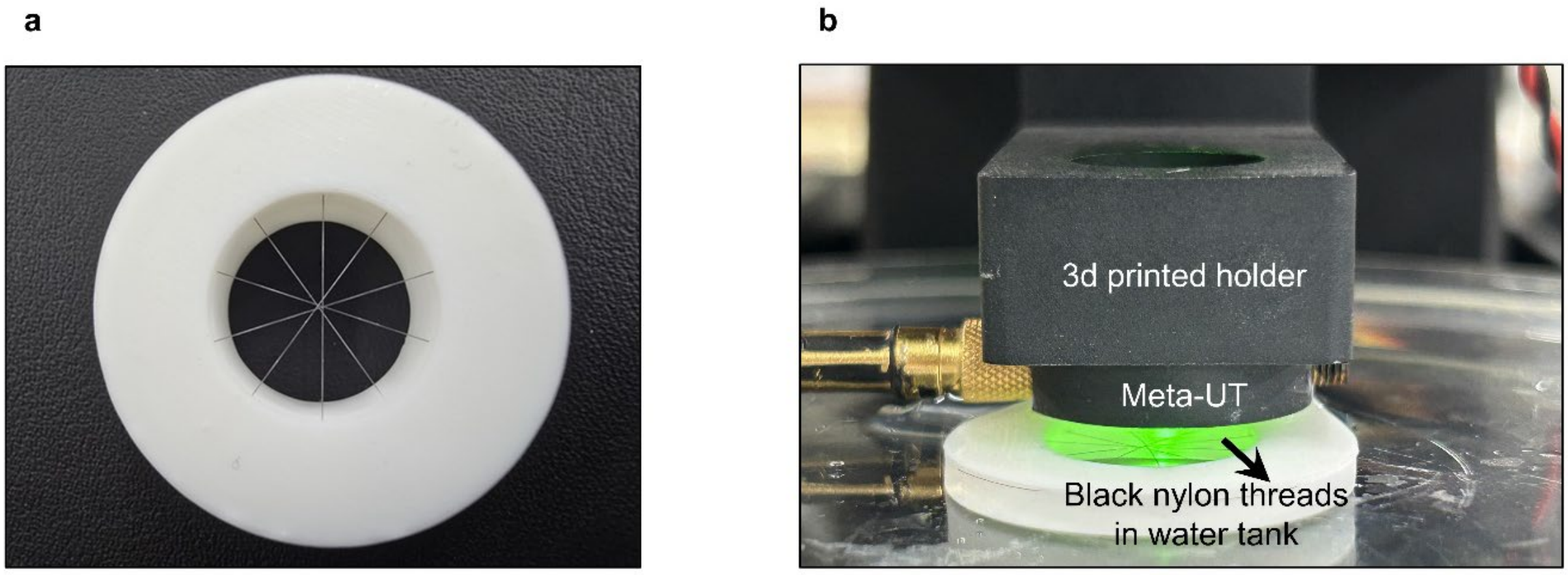

Figure S14. Photographs of the nylon thread phantom (a) and the experimental setup (b).

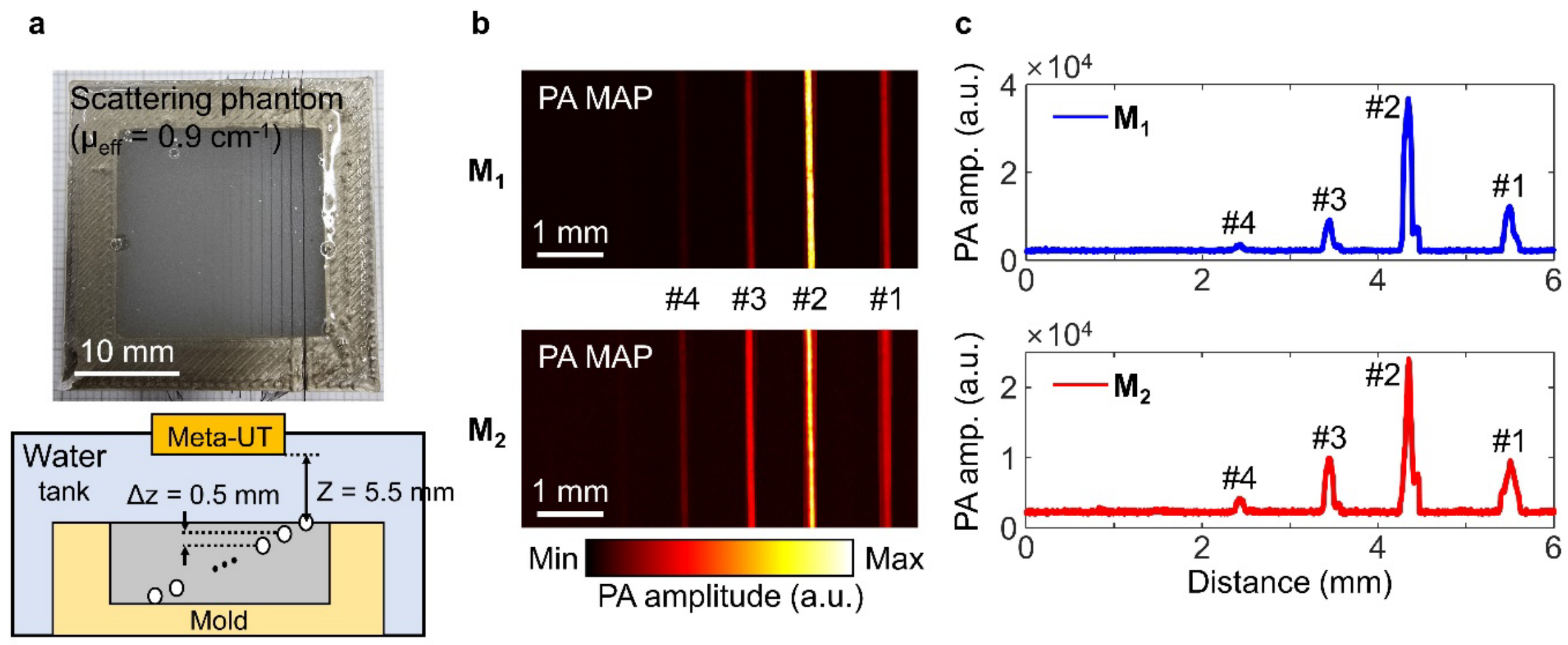

Figure S15. Characterization of dual-focus meta-PAM under scattering conditions. (a) Photograph and schematic of the scattering phantom experimental setup. Nylon threads were embedded in scattering media with an effective scattering coefficient of 0.9 cm^{-1}, starting at a depth of 5.5 mm from the meta-UT and spaced at 0.5 mm intervals. (b) PA MAP image of axially positioned nylon threads embedded in scattering media for both M_1 and M_2 modes. Thread #2 is positioned at M_1 focal plane, whereas thread #4 is positioned at M_2 focal plane. (c) Line PA amplitude profiles for each mode, showing the variation in PA amplitude contrast of nylon threads at different depths.

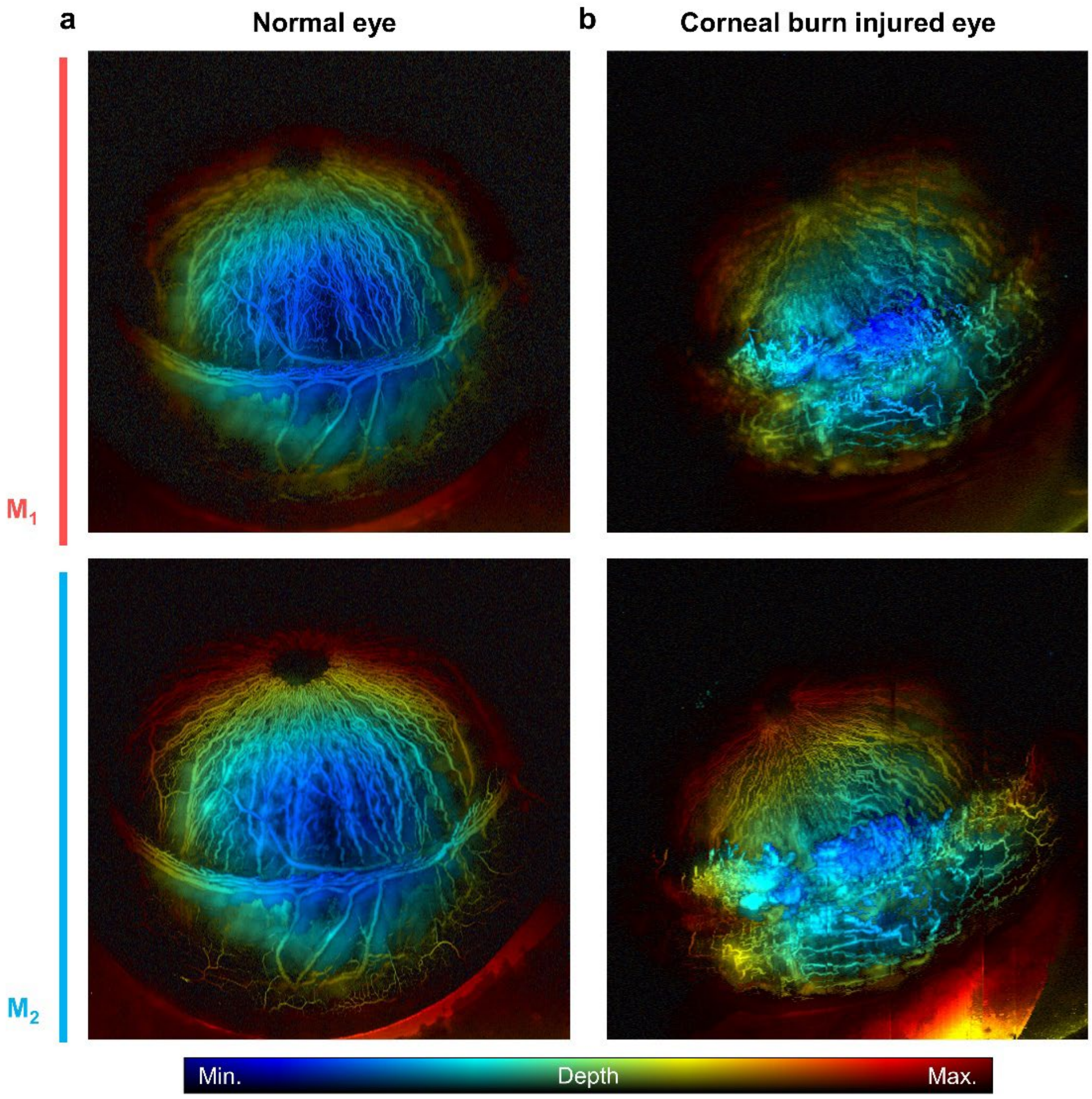

Figure S16. Depth-encoded photoacoustic maximum amplitude projection (PA MAP) images of a rat eye in vivo. M_1 represents the short-focus condition, while M_2 represents the long-focus condition. (a) Normal rat eye. (b) Corneal burn-injured eye. Blue indicates shallow depths, while red represents deeper depths.

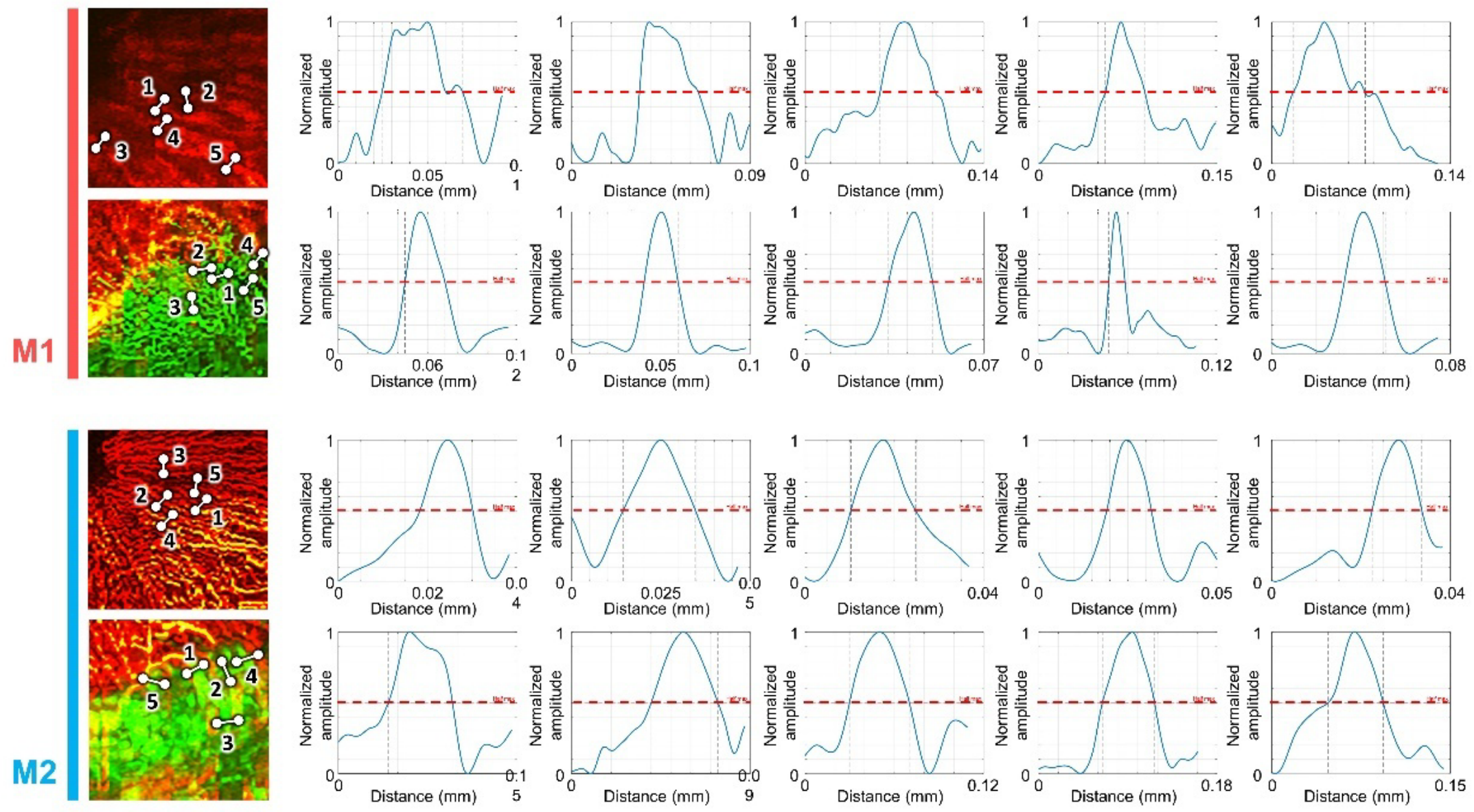

Figure S17. Vessel diameter quantification under the M_1 and M_2 focal modes. PA amplitude line profiles across iris and CNV vessels were extracted, and vessel diameters were determined from the full width at half maximum (FWHM) of the corresponding profiles.

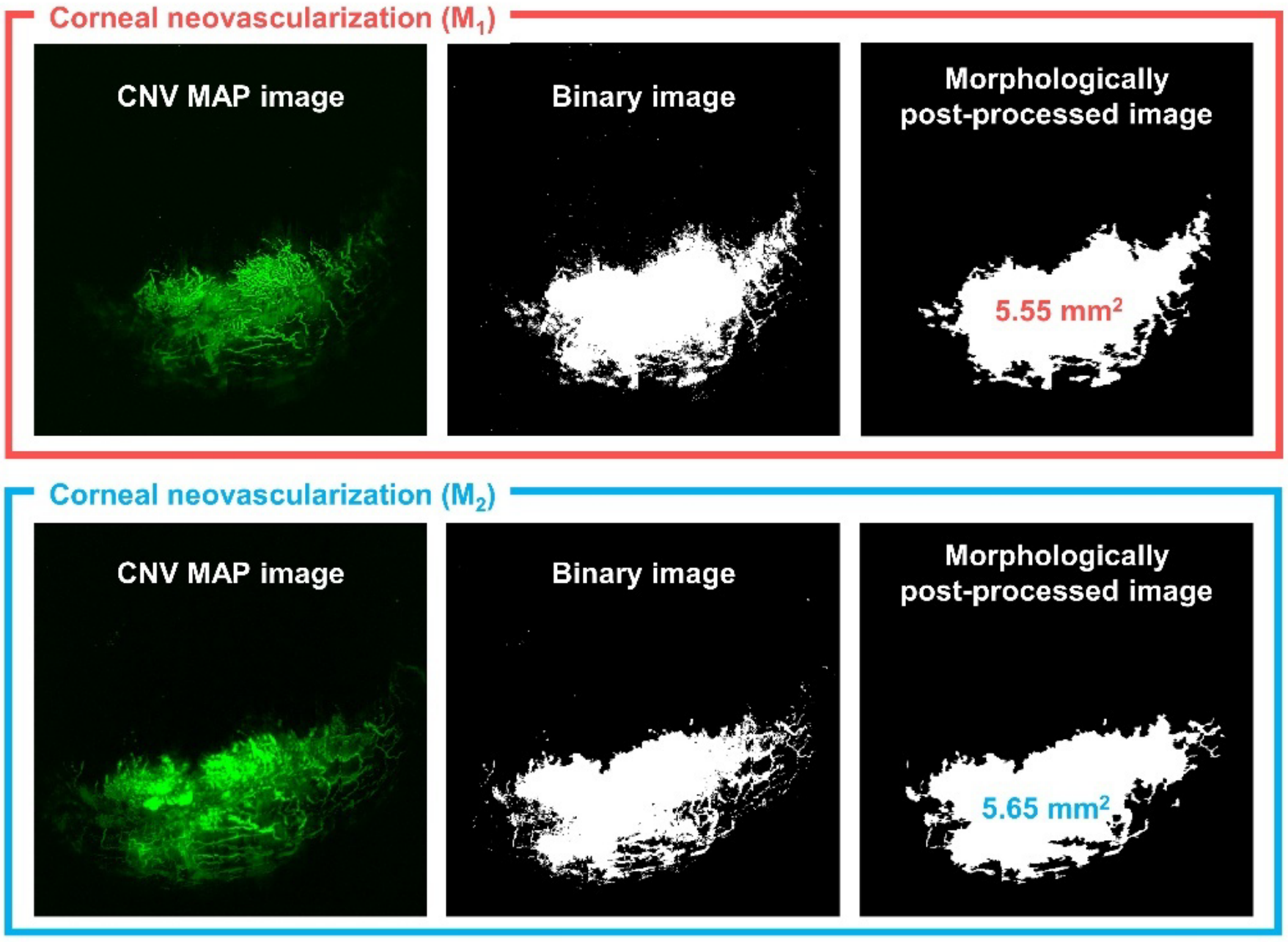

Figure S18. Workflow for quantifying corneal neovascularization (CNV) lesion area. Blood vessels in the corneal layer were selectively extracted and visualized in green. The CNV MAP image was converted to a binary image, followed by morphological post-processing (small-object removal, hole filling, and closing) to obtain clean, continuous vascular structures. The lesion area was then quantified by counting the pixels in the processed mask and multiplying by the pixel area.

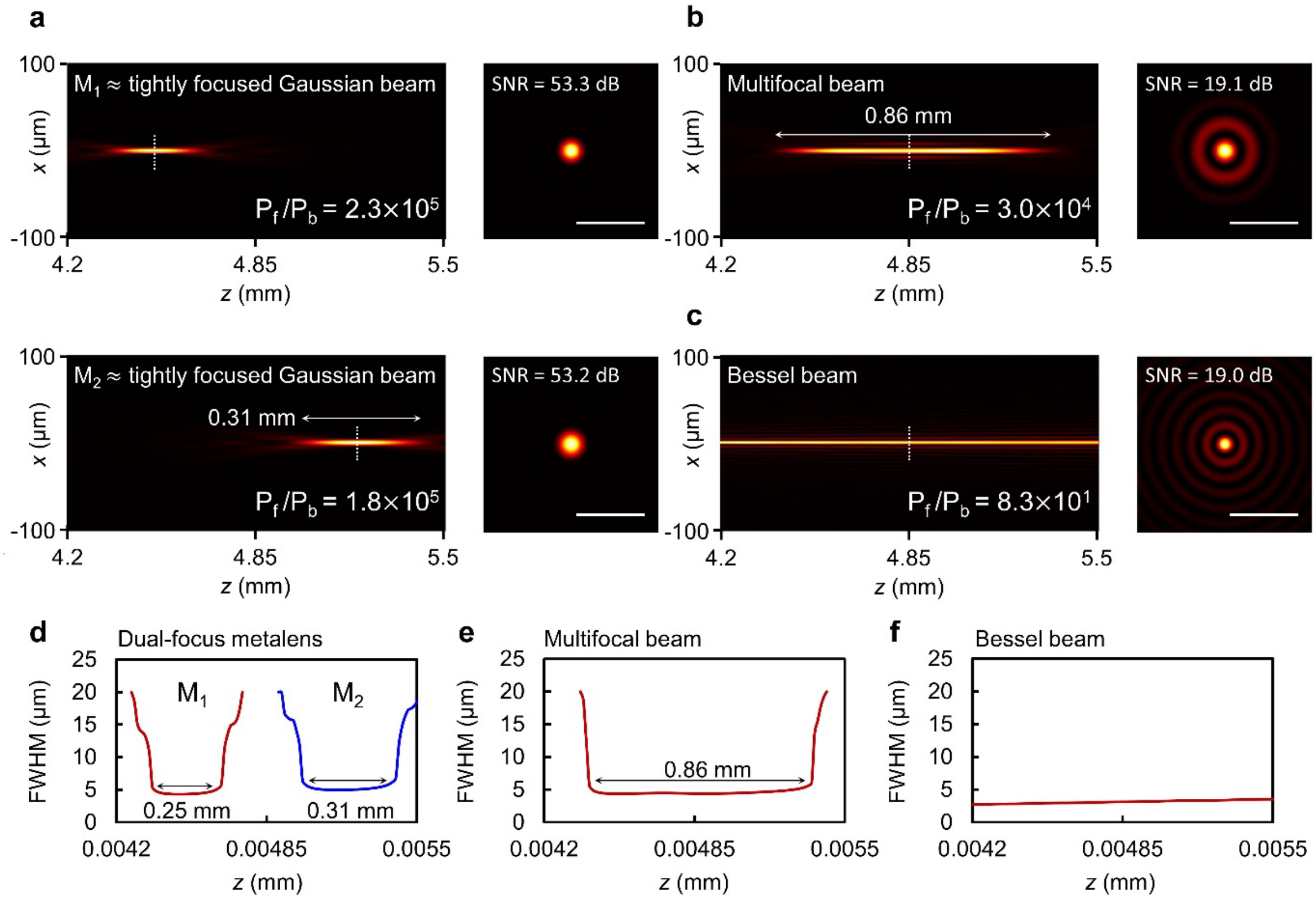

Figure S19. Comparison of multiple approaches based on *xz* and focal-plane *xy* profiles, SNR, and focal intensity ratio. Simulated *xz* intensity distributions (left) and focal-plane *xy* intensity maps (right) for (a) the dual-focus, (b) the multifocal needle-beam, and (c) the Bessel-beam approach (Scale bars: 10 μm). Full width at half maximum (FWHM) as a function of *z* for (d) the dual-focus, (e) the multifocal needle-beam, and (f) the Bessel-beam approach.

The needle-beam design was realized by introducing m = 57 multiple focal points. First, the hyperbolic phase profiles corresponding to each focal position were calculated. In this case, the focal points were uniformly distributed from 4.45 mm to 5.25 mm. Each phase profile was then multiplied by a binary random localization function, where the m = 57 number of localization functions were designed to be mutually non-overlapping. The final phase mask was obtained by summing all products of the hyperbolic lens phases and their respective random localization functions. To further refine the beam profile, we applied a point-spread optimization method to optimize the random localization, thereby generating a needle beam with an extended depth of focus (DOF) and uniform axial intensity distribution. The Bessel-beam was designed by imposing an axicon-like radial phase $\phi(r) = kr\sin\theta$, where $NA_{bessel} = \sin\theta = 0.067$.

The focal intensity ratio was defined as P_f/P_b, where P_f is the mean intensity within the focal-spot region and P_b is the mean background intensity measured in an annular region outside the focal spot. The SNR was calculated using the same definition as $\mathrm{SNR}=20\log((P_f - P_b)/\sigma_b)$, where σ_b is the standard deviation of the background intensity. The focal-spot region was defined as a circular region centered at the peak intensity with a radius r_f determined from the diffraction-limited spot size ($r_f = 0.5\lambda/\mathrm{NA}$). The background was defined as an annular region outside the focal spot ($2r_f \le r \le 4r_f$) to minimize contamination from the focal tail.

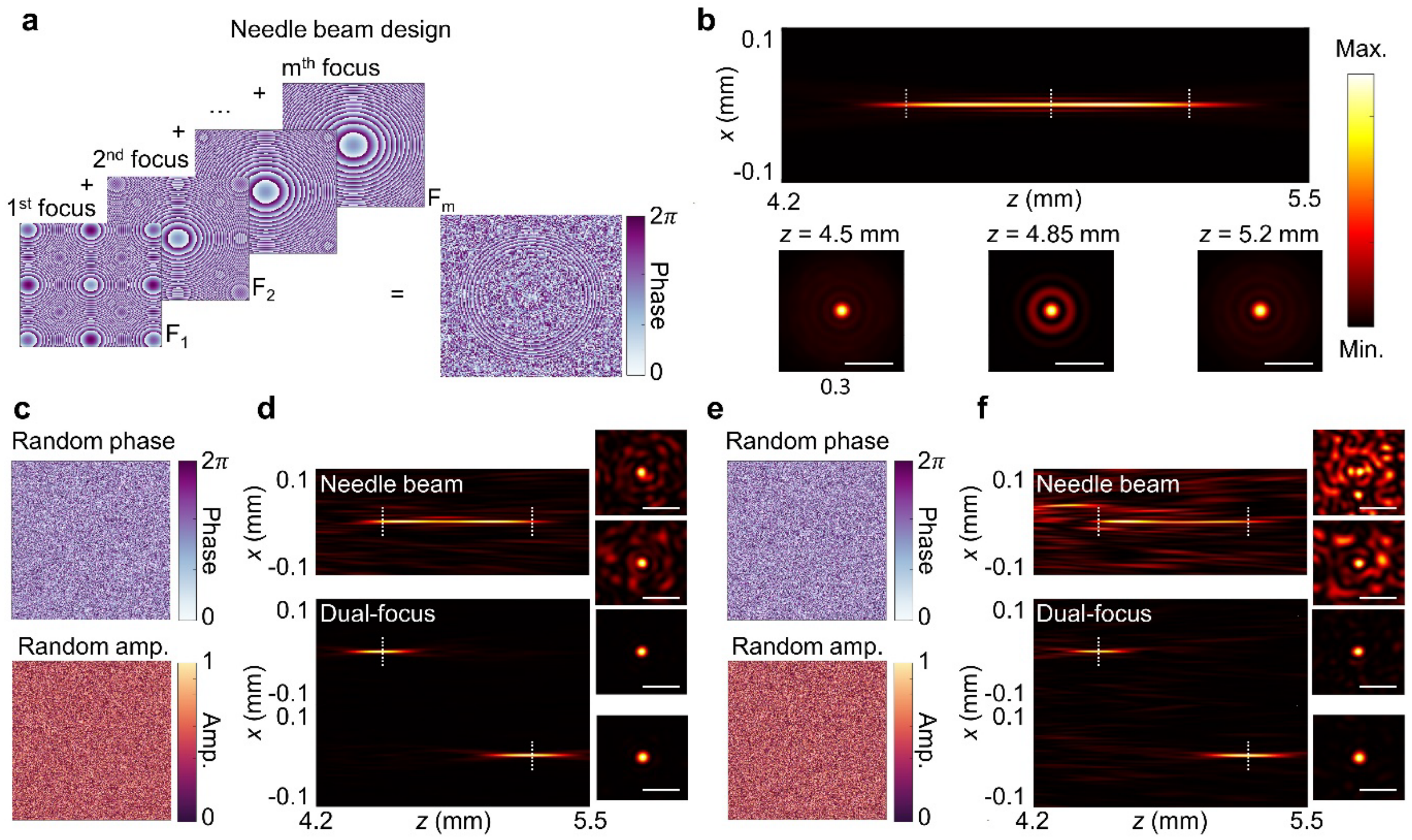

Figure S20. Comparison between needle beam and dual-focus approach. (a) Design method of needle beam design. Hyperbolic lens phase for each focus is multiplied with a distinct binary matrix and are added to produce the final phase map F_1, F_2, and F_m corresponds to focal length of each hyperbolic lens phase. (b) Simulated *xz* intensity profile of the optimized needle beam lens and *xy* focal intensity distribution for each *z*. The needle beam is optimized to achieve long depth of focus along 4.5 mm to 5.2 mm to match the dual-focus metalens focal length (Scale bars: 10 μm) (c,e) Introduced random phase and amplitude map to the needle beam and dual-focus metalens. Random variations were applied to each pixel of the complex field, with slight spatial correlation introduced to form clustered random patterns. Higher random variations are applied at e. (d,f) Simulated *xz* and *xy* focal intensity profile (4.5 mm and 5.2 mm) of needle beam and dual-focus metalens propagating through the random surface. (Scale bars: 10 μm)

Imaging modality	Lateral resolution	Imaging depth coverage	Imaging depth	Scanning Method	Sensitivity	Remarks	Reference
Ultrasound Localization Microscopy (ULM)	20-50 μm	>10 mm	>10 mm	Plane-wave angle compounding	High with MBs	Requires exogenous MBs, and sensitivity declines over time as MBs clear. Long acquisition time	[48-50]
Optical Microscopy	<1 μm	<10 μm	<0.3 mm	Galvanometer mirror scan	High in superficial layers	Excellent superficial imaging; not suitable for intact ocular vasculature	[51,52]
Optical Coherence Tomography (OCT)	5-10 μm	~ 0.4 mm	1-2 mm	Galvanometer mirror scan	High for structural imaging	OCT angiography (OCTA) visualizes flow, but has limited sensitivity to slow flow.	[53,54]
Conventional PAM	3-4 μm	~ 0.4 mm	>2 mm	2D motorized stage	High with intrinsic absorption contrast	Functional & vascular imaging.	[55]
Dual-focus Meta-PAM (This work)	3-4 μm	~1.2 mm	>2 mm	2D motorized stage	High with intrinsic absorption contrast	Functional & vascular imaging.	Our work

Figure S21. Comparison of optical imaging modalities for the anterior segment.

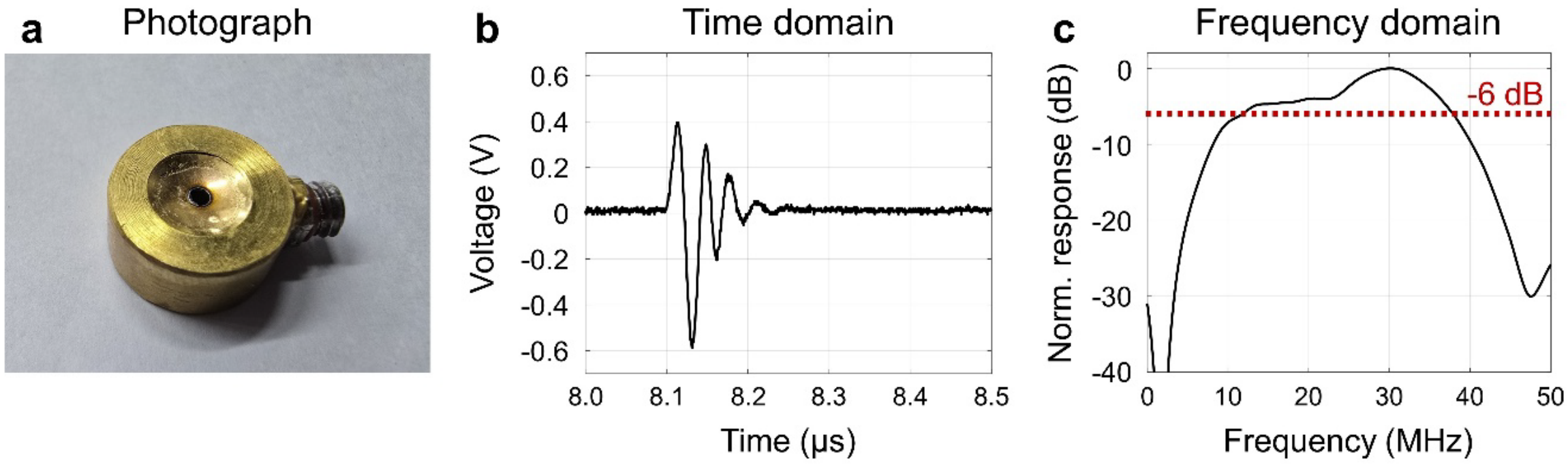

Figure S22. Fabricated ring-shaped ultrasound transducer. (a) Photograph of the transducer, which has a central hole designed for integration with a meta-lens. (b) Time-domain plot of the ultrasound signal acquired via pulse-echo testing. (c) Frequency-domain analysis using the fast Fourier transform (FFT). The center frequency is 25 MHz, and the −6 dB bandwidth is calculated to be 106%.
